# Socio-geographical disparities in cardiometabolic multimorbidity in Sweden: an Intersectional Multilevel Analysis of Individual Heterogeneity and Discriminatory Accuracy (I-MAIHDA)

**DOI:** 10.1186/s12939-025-02684-z

**Published:** 2025-11-04

**Authors:** Kanya Anindya, Juan Merlo, Lars Lind, Lars Weinehall, Marcus Bendtsen, Tomas Jernberg, Maria Rosvall, Nawi Ng

**Affiliations:** 1 School of Public Health and Community Medicine, Institute of Medicine, Sahlgrenska Academy, University of Gothenburg, Gothenburg, Sweden; 2 Unit for Social Epidemiology, Faculty of Medicine, Lund University, Malmö, Sweden; 3 Centre for Primary Health Care Research, Region Skåne, Malmö, Sweden; 4 Department of Medical Sciences, Uppsala University, Uppsala, Sweden; 5 Department of Epidemiology and Global Health, Umeå University, Umeå, Sweden; 6 Department of Health, Medicine and Caring Sciences, Linköping University, Linköping, Sweden; 7 Department of Clinical Sciences, Danderyd Hospital, Karolinska Institute, Stockholm, Sweden; 8 Social Medicine, Region Västra Götaland, FoUUI, Regionhälsan, Sweden

**Keywords:** Multimorbidity, Cardiovascular disease, Diabetes, Intersectional framework, Social inequity

## Abstract

**Background:**

Mapping social and geographical disparities in health outcomes helps to identify vulnerable groups that should be targeted for intervention. This study aims to assess the disparities in cardiometabolic multimorbidity, the presence of at least two cardiometabolic diseases (CMD), including type 2 diabetes, heart disease, and stroke, among middle-aged adults across socio-geographical intersectional strata in Sweden.

**Methods:**

This cross-sectional study used the first examination (2013–2018) of the Swedish CArdioPulmonary bioImage Study (SCAPIS), with a total sample of 29,093 individuals aged 50–64 years living in six areas in Sweden (Gothenburg, Linköping, Malmö/Lund, Stockholm, Umeå and Uppsala). Cardiometabolic multimorbidity was identified based on self-reported information, the National Patient Register, the Swedish Prescribed Drug Register, and examination of glycaemic status. We constructed ninety-six socio-geographical intersectional strata based on the combination of age (50–59/60–64 years), sex (females/males), education (low/high), country of birth (Swedish/foreign-born), and six geographical areas. Intersectional multilevel analysis of individual heterogeneity and discriminatory accuracy (I-MAIHDA) was used to map the predicted prevalence of cardiometabolic multimorbidity and quantify the variance partition coefficient (VPC) and area under the receiver operating characteristic curve (AUC).

**Results:**

24.4% of the participants had one CMD, and 2.8% had cardiometabolic multimorbidity. Across the six areas, strata of 60–64-year-old males with low education, irrespective of country of birth, had the highest prevalence of cardiometabolic multimorbidity. The highest prevalence was observed in 60–64-year-old foreign-born males with low education in Gothenburg (12.4%, 95% CI 7.1–19.3) and in 60–64-year-old Swedish-born males with low education in Malmö (8.6%, 95% CI 6.3–11.3). The VPC was high (15.0%, 95% CI 10.5–21.1), indicating the importance of intersectional strata in explaining disparities in cardiometabolic multimorbidity, with an AUC of 0.71 (95% CI 0.70–0.73).

**Conclusions:**

By applying an intersectionality framework, our study provides a more nuanced map of disparities in cardiometabolic multimorbidity to inform preventive strategies aligned with proportionate universalism and precision of public health. The heterogeneity and discriminatory accuracy measures suggest the need for universally tailored intervention strategies to prevent cardiometabolic multimorbidity.

**Supplementary Information:**

The online version contains supplementary material available at 10.1186/s12939-025-02684-z.

## Introduction

Cardiometabolic multimorbidity, typically defined as the co-existence of at least two cardiometabolic diseases (CMD), including type 2 diabetes (T2D), heart disease and stroke, is one of the most prevalent multimorbidity clusters in the general population [1, 2]. A study based on the Swedish Twin Registry estimated that 4.7% of dementia-free individuals aged 60 years and older had cardiometabolic multimorbidity in 2016 [3].

The burden of CMD is unequally distributed across social dimensions, such as age, sex, socioeconomic status, and migration status [4–7]. These disparities can be explained by several interrelated mechanisms: biological ageing, sex-based physiological and behavioural differences, socioeconomic barriers to care, and migration-related stressors [4–6]. Geographical variation also exists, with higher rates of cardiovascular disease (CVD) observed in northern municipalities of Sweden [8]. The intersection of these factors creates cumulative vulnerability to the progression of cardiometabolic multimorbidity. Focusing on this accumulation of disease is important, as it reflects social disparities in care that can further widen inequalities beyond single CMD [9].

Conventional epidemiological studies, however, typically rely on estimating the average association (e.g., odds ratio) between a single social dimension and disease risk [10–12]. This traditional approach has been questioned for two primary reasons. *First*, the risk of CMD is not shaped by a single social dimension in isolation but by a complex intersection of multiple social dimensions. Public health epidemiology increasingly applies *intersectionality theory* [13], which views social dimensions (e.g., sex/gender, race/ethnicity, and migration status) as interlocking factors that shape distinct risk profiles [13–17]. *Second*, the reliance on a measure of average association leads to ‘tyranny of the averages’ [18, 19], assuming the same average risk for all individuals within a group. This approach overlooks individual heterogeneity within groups and how it may overlap between groups [15, 20]. Therefore, studies are encouraged to assess the discriminatory accuracy (DA) of group disparities [21], i.e., how well social dimensions distinguish individuals with the disease from those without the disease [15, 19]. As Merlo et al. (2017) argue, understanding DA is crucial, as interventions based on measures of association with low DA can stigmatise ‘high-risk’ groups and underestimate the risk of ‘low-risk’ groups [19, 20, 22]. Integrating an intersectional approach and DA aligns with precision of public health [20, 23, 24], “*the right intervention to the right population at the right time*” [24, p. 398] and helps determine whether preventive strategies should be universal, proportionately universal, or targeted [25, 26].

Intersectional Multilevel Analysis of Individual Heterogeneity and Discriminatory Accuracy (I-MAIHDA) may address these criticisms [15, 27, 28]. MAIHDA has been applied in geographical [29–31] and healthcare epidemiological studies [16, 22, 28, 32–35] and offers a robust method for assessing health disparities consistent with ‘proportionate universalism’ and ‘precision public health’ [15, 27]. I-MAIHDA quantifies the within- and between-stratum variance and distinguishes additive from interactive (residual) effects [15, 36, 37]. This study aims to (i) provide an intersectional mapping of the prevalence of cardiometabolic multimorbidity across intersectional strata, (ii) quantify the discriminatory accuracy of the intersectional strata, and (iii) assess the extent to which a potential intersectional association between the intersectional strata and cardiometabolic multimorbidity is due to the interaction of the effects of the dimensions defining the strata. We hypothesise that there is a geographical disparity in cardiometabolic disease and multimorbidity in Sweden, with a higher prevalence in northern areas, and that this disparity is patterned by the intersection of age, sex, socioeconomic position, and country of birth.

## Methods

### Data sources and study population

This cross-sectional study utilised population-based data from the first examination of the Swedish Cardiopulmonary Bioimage Study (SCAPIS), conducted between November 2013 and December 2018 [38]. SCAPIS, a prospective community-based cohort, was collected in areas surrounding six university hospitals in Sweden (Gothenburg, Linköping, Malmö/Lund, Stockholm, Umeå and Uppsala). SCAPIS invited 59,909 randomly selected individuals aged 50–64 years from the Swedish population register, of whom 30,154 participated (50.3%) [38]. This study integrated the SCAPIS dataset with the Swedish Prescribed Drug Register for drug prescriptions and the National Patient Register (NPR) for inpatient and outpatient information. Only participants who consented to link their data to the registers were included in the analysis (*n* = 30,030). Among these, individuals with missing values were excluded (3.1%), resulting in a final sample of 29,093 participants. The number of missing values for each variable is provided in Table S1 (Supplementary material 1), along with the sample flowchart (Figure S1).

### Variables

#### Outcome variables

CMD was defined as T2D, heart disease (including coronary heart disease and heart failure), and stroke. The co-existence of two or three CMDs in an individual was considered cardiometabolic multimorbidity. CMD was identified based on multiple resources to reduce the potential recall bias. These included: (1) self-reported information available in the SCAPIS database; (2) CMD diagnosis based on the International Classification of Diseases (ICD) codes in the NPR; (3) Anatomical Therapeutic Chemical (ATC) codes for glucose-lowering medications in the drug register; and (4) glycaemic status assessment identified during the SCAPIS interview. Participants were classified as having CMD(s) if the condition was identified in at least one data source. The list of ICD and ATP codes used to identify CMD is available in Table S2, while Table S3 details the variables’ measurements (Supplementary material 1).

The total number of CMDs was calculated for each participant, ranging from zero (no CMD) to three CMDs. Subsequently, two outcome variables were formed in the analyses: (1) any CMD (no CMD vs. having at least one CMD) and (2) cardiometabolic multimorbidity (no/single CMD vs. ≥ 2 CMDs).

#### Social and geographical dimensions

We selected social dimensions based on prior research [6, 39, 40], which demonstrated a strong association with CMD, and the intersectional identities framework by Beard et al., which incorporates ascribed, achieved, and geopolitical identities [41]. Social dimensions included in this study were age, sex, educational attainment, and country of birth. The chosen variables were used for constructing intersectional strata (see below). Some potentially relevant variables, such as financial situation and employment [42], were not included in the model to reduce the potential reverse causation between exposures and outcomes due to the cross-sectional nature of the dataset. The number of dimensions chosen in the study also considered the minimum cell size.

Sex consists of females and males, while age was grouped into 50–59 and 60–64 years. Participants’ self-reported educational level was recorded as not completed primary school, primary school (maximum nine years), secondary school/high school/vocational training, and university or college degree. Based on these responses, educational level was dichotomised into low (secondary school/lower) and high (university or college degree). Country of birth was grouped as Swedish-born and foreign-born, regardless of the parents’ country of birth, following the definition used by Statistics Sweden [43]. The geographical dimension was assessed based on the six examination areas of SCAPIS. The six areas include Gothenburg, Linköping, Malmö/Lund, Stockholm, Umeå and Uppsala.

#### Intersectional strata

We constructed an intersectional variable in two steps. First, we used all the possible combinations of the four social dimensions, resulting in 16 social strata (2 × 2 × 2 × 2). Second, we added the geographical dimension by considering the unique combination of the six areas and the 16 social strata, resulting in 96 socio-geographical strata (16 social strata × 6 areas). The sample size for each stratum is available in Tables S4–S5 (Supplementary material 1).

### Statistical analysis

Descriptive statistics were used to summarise participants’ characteristics across CMD status. We estimated the age-sex-standardised prevalence of CMD status using population data from 2015 Statistics Sweden [44]. Subsequently, this study applied I-MAIHDA to assess disparities across social and geographical strata. Following previous studies [27, 28, 34], we performed I-MAIHDA using three sets of multilevel logistic regression models.

#### Model 1: simple intersectional model

Model 1 is a random intercept null model consisting of two different sub-models: Model 1A nested individuals (at level 1) within 16 social strata (at level 2), and Model 1B nested individuals (at level 1) within 96 socio-geographical strata (at level 2). The models decomposed the total individual variance in the outcomes (i.e., any CMD or cardiometabolic multimorbidity) into its within and between strata components.

Model 1 calculated the predicted prevalences of outcomes and 95% confidence intervals (CI) across 16 social strata and 96 socio-geographical strata. The stratum prevalence indicates the proportion of individuals having the outcome at a given time, which can also represent the absolute risk. The predicted prevalences were obtained by transforming the predicted logit (log-odds) of having the outcomes in stratum *j* (Eq. 1),

1 $$\:{\pi\:}_{j}={{logit}^{-1}(\beta\:}_{0}+\:{u}_{j})$$

$$\:{u}_{\text{j}}\:\sim\:N\:(0,\:{{\sigma\:}^{2}}_{u})$$

where *π*_*j*_ denotes the predicted probability of having the outcome in stratum *j*, *β*_*0*_ denotes the intercept, and *u*_*j*_ is the random effect for stratum *j* (*j* = 1,…,16 for Model 1A and *j* = 1,…,96 for Model 1B).

Model 1 also provided information on the relevance of the intersectional strata for understanding individual inequalities in the outcome, commonly called the intersectional ‘general contextual effect’ (GCE) [15, 27, 45]. GCE can be quantified using the variance partition coefficient (VPC). VPC indicates the proportion of total individual variation in the outcome attributable to the between-stratum level (Eq. 2), where *σ* ^*2*^_*u*_ refers to between-stratum variance (var(*u*_*j*_)) and within-stratum-between-individual variance was set to $\:\frac{{\pi\:}^{2}}{3}\approx\:\:$3.29 for logistic distribution, as described by Goldstein et al. [46]. The VPC in perfect hierarchical data is a measure of homogeneity, informing on the clustering or intra-strata correlation of the outcome, with values between 0% and 100%. Following previous studies [22, 28, 47], VPCs were classified into small (≤ 5%), moderate (5–10%), high (10–20%) or very high (≥ 20%).

2 $$\:VPC=\frac{{{\sigma\:}^{2}}_{u}}{{{\sigma\:}^{2}}_{u}+\:\frac{{\pi\:}^{2}}{3}}$$

Furthermore, Model 1 also evaluated DA, measured by the area under the receiver operating characteristic curve (AUC). The AUC assesses the accuracy of social/socio-geographical strata in correctly classifying individuals with and without the outcomes. The AUC ranges between 0.5 (no discrimination) and 1.0 (perfect discrimination). While it has been suggested not to categorise AUC values into ‘good/bad’ threshold [27], this study follows the classification by Hosmer and Lemeshow for easier interpretation [48]: no discrimination (AUC = 0.5), poor (0.5 < AUC < 0.7), acceptable (0.7 ≤ AUC < 0.8), excellent (0.8 ≤ AUC < 0.9), and outstanding discrimination (AUC ≥ 0.9). From a public health perspective, an AUC value greater than 0.7 is considered relevant [33].

The information from the VPC and AUC is similar and complementary; see elsewhere for further explanation [33]. When the size of the intersectional strata is similar (balanced data), the VPC and the AUC give similar information, and both procedures can be used to measure DA and GCE [27]. However, when the size of intersectional strata is very different (unbalanced), the VPC informs the GCE, and the AUC informs the DA, so they provide complementary information [29]. Thus, it is also important to consider the absolute number of cases in each stratum. The VPC and AUC obtained from Model 1A and Model 1B were compared to assess which model had better GCE and DA. A sub-model with a higher value of AUC was chosen for the next step of analyses (Model 2 and Model 3), while a sub-model with a lower AUC was presented in the sensitivity analysis.

#### Model 2: partially-adjusted intersectional model

Model 2 expanded the previous model (Model 1A or 1B) by adjusting *one* variable (*x*_*j*_) used to construct the intersectional strata at a time in the fixed part of the model (Eq. 3).

3 $$\:{\pi}_{j}={{logit}^{-1}(\beta\:}_{0}+\:{\beta\:}_{1}{x}_{j}+\:{u}_{j})$$

$$\:{u}_{\text{j}}\:\sim\:N\:(0,\:{{\sigma\:}^{2}}_{u})$$

Model 2 assessed to what degree the different variables used to construct the intersectional strata contributed to the between-stratum variance obtained in Model 1. This was done by calculating the proportional change in between-stratum variance (PCV, Eq. 4), where *σ* ^*2*^_*u(Model 1)*_ refers to between-stratum variance for the selected Model 1 (Eq. 1) and *σ* ^*2*^_*u(Model 2)*_ refers to between-stratum variance for Model 2 (Eq. 3). Model 2 was estimated four times (if Model 1A was selected) or five times (if Model 1B was selected), according to the numbers of variables used to construct intersectional strata.

4 $$\:PVC=\:\frac{{{\sigma\:}^{2}}_{u\left(\text{M}\text{o}\text{d}\text{e}\text{l}\:1\right)}\:-{{\:\sigma\:}^{2}}_{u\left(\text{M}\text{o}\text{d}\text{e}\text{l}\:2\right)}\:}{{{\sigma\:}^{2}}_{u\left(\text{M}\text{o}\text{d}\text{e}\text{l}\:1\right)}}$$

#### Model 3: intersectional interaction model

Model 3 simultaneously included *all variables* used to construct the intersectional strata in the fixed part of the model (Eq. 5). It decomposes the prevalence or absolute risk into two parts: (1) the absolute risk due to the main effect (ARDM, the fixed part of the model: $\:{\beta\:}_{0}+\:{\beta\:}_{1}{x}_{1j}+{\dots\:+\beta\:}_{5}{x}_{5j}$) and (2) the absolute risk due to interaction effect (ARDI, the stratum random effect: *u*_*j*_) [49]. A positive interaction effect (*ARDI*_*j*_ >0) indicates that the intersectional stratum has a higher predicted prevalence than expected from the main effects alone. In comparison, a negative interaction effect (*ARDI*_*j*_ < 0) suggests a lower prevalence than expected [49]. Odds ratios (ORs) were estimated from the fixed effects in Model 2 and Model 3.

5 $$\:{\pi\:}_{j}={{logit}^{-1}(\beta\:}_{0}+\:{\beta\:}_{1}{x}_{1j}+{\dots\:+\beta\:}_{5}{x}_{5j}+\:{u}_{j})$$

$$\:{u}_{\text{j}}\:\sim\:N\:(0,\:{{\sigma\:}^{2}}_{u})$$

#### Sensitivity analyses

Four sensitivity analyses were conducted. First, we estimated differences in CMD prevalence when using multiple data sources (self-reports, register data, and glycaemic status assessments) compared with only self-reported information, to quantify the extent of over- or underestimation that occurs when relying solely on self-reported information. Second, we estimated the predicted probabilities of T2D and CVD (heart disease and stroke) separately to assess whether the pattern of vulnerability was consistent with that observed in the main model. Third, we replaced educational level with employment status (employed/unemployed) to define the intersectional strata. Fourth, we refined the classification of Swedish- and foreign-born individuals by considering parents’ country of birth. Individuals born outside Sweden with both foreign-born parents were classified as ‘foreign-born’. Those born in Sweden with both foreign-born parents were classified as ‘second-generation migrants’. Individuals born outside or within Sweden with at least one Swedish-born parent were classified as ‘Swedish-born’.

I-MAIHDA was performed in MLwiN 3.02, called from Stata/SE 18.0 (StataCorp LLC, College Station, TX), using the *runmlwin* command [50]. We used the Restricted Generalised Least Squares (RIGLS) method to obtain starting values for the final Markov Chain Monte Carlo (MCMC) estimations. The MCMC method provided the posterior distributions of the parameters, which were then used to estimate measures of association and variance [28, 51]. The study is reported according to the Strengthening the Reporting of Observational Studies in Epidemiology (STROBE) guideline.

## Results

Overall, the mean age of the participants was 57.1 (SD 4.4) (Table 1). Of the 29,093 participants, 51.7% were female, 45.1% attained tertiary education, and 83.9% were Swedish-born. The age-sex-standardised prevalences of having at least one CMD(s) and multimorbidity were 24.4% and 2.8%, respectively. Stockholm had the highest proportion of individuals with single CMD (28.7%), while Umeå (3.0%) and Malmö (3.6%) had a higher prevalence of cardiometabolic multimorbidity.

**Table 1:** Descriptive statistics of respondents’ cardiometabolic disease status

Variables	All^1^	Cardiometabolic disease status^2^		
		No CMD	Single CMD	Multimorbidity
	*N* (%)	*n* (%)	*n* (%)	*n* (%)
**Total**	29,093 (100.0)	21,177 (72.8)	7109 (24.4)	807 (2.8)
Age, mean (sd)	57.1 (4.4)	56.68 (4.3)	57.89 (4.3)	59.40 (3.9)
Age				
50–59 years	19,342 (66.5)	14,796 (76.5)	4185 (21.6)	361 (1.9)
60–64 years	9751 (33.5)	6381 (65.4)	2924 (30.0)	446 (4.6)
Sex				
Female	15,032 (51.7)	11,533 (76.7)	3250 (21.6)	249 (1.7)
Male	14,061 (48.3)	9644 (68.6)	3859 (27.4)	558 (4.0)
Educational attainment				
Tertiary	13,133 (45.1)	10,139 (77.2)	2750 (20.9)	244 (1.9)
Secondary/lower	15,960 (54.9)	11,038 (69.2)	4359 (27.3)	563 (3.5)
Country of birth				
Swedish-born	24,403 (83.9)	17,969 (73.6)	5794 (23.7)	640 (2.6)
Foreign-born	4690 (16.1)	3208 (68.4)	1315 (28.0)	167 (3.6)
Area				
Göteborg	6133 (21.1)	4598 (75.0)	1371 (22.4)	164 (2.7)
Linköping	4901 (16.8)	3671 (74.9)	1112 (22.7)	118 (2.4)
Malmö	5954 (20.5)	4388 (73.7)	1353 (22.7)	213 (3.6)
Stockholm	4961 (17.1)	3405 (68.6)	1426 (28.7)	130 (2.6)
Umeå	2393 (8.2)	1751 (73.2)	570 (23.8)	72 (3.0)
Uppsala	4751 (16.3)	3364 (70.8)	1277 (26.9)	110 (2.3)

Notes

^1)^ Column percentages

^2)^ Row percentages. Cardiometabolic diseases are type 2 diabetes, heart disease (CHD and heart failure), and stroke

CMD multimorbidity was defined as a condition in which at least two cardiometabolic diseases are present in a person. The overall prevalence (%) of CMD status were age-sex-standardised

**Fig. 1:**
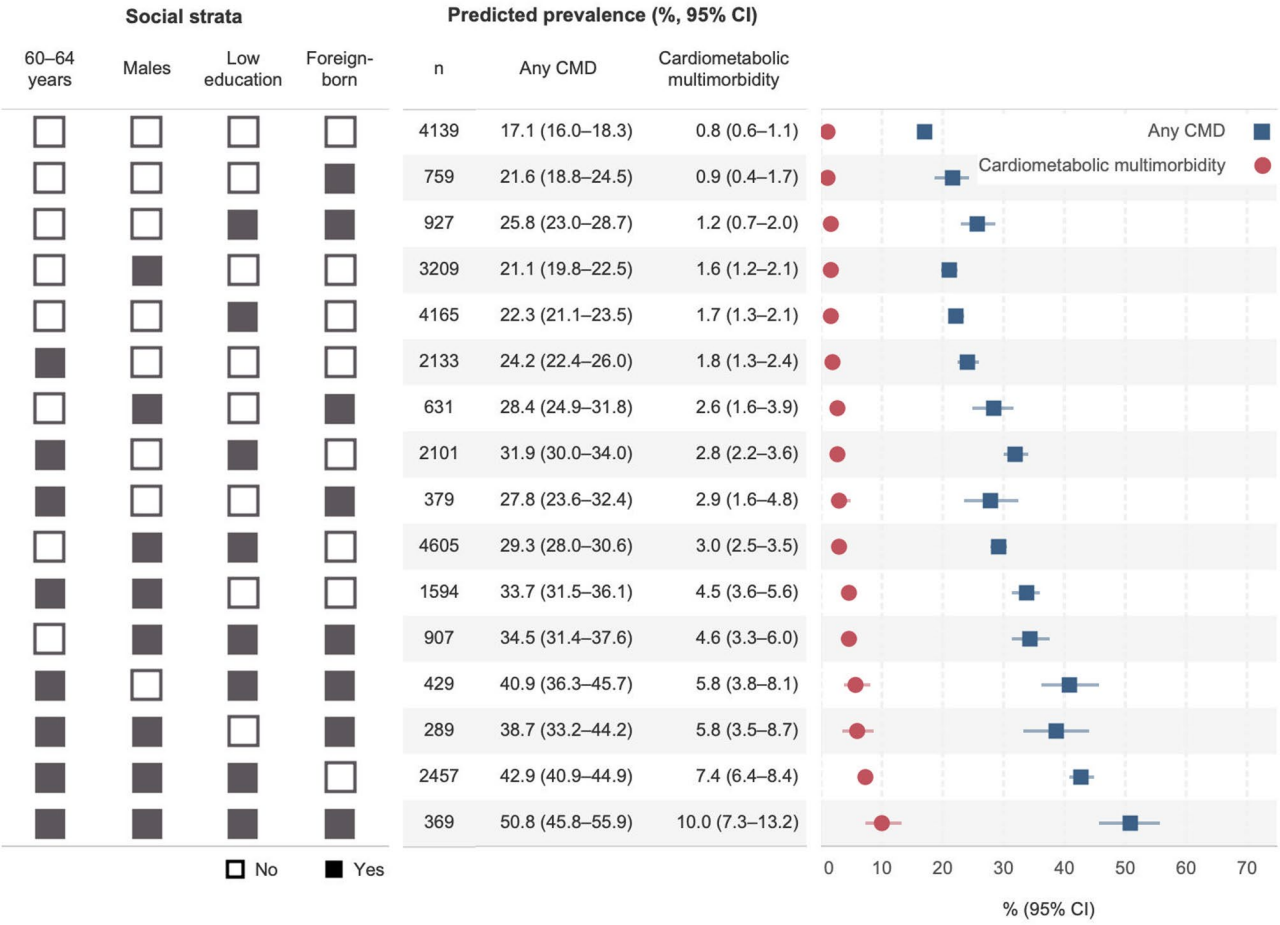
Predicted prevalence of any cardiometabolic disease and cardiometabolic multimorbidity, by social strata. Notes: CMD: Cardiometabolic disease; CI: Confidence interval. Predicted prevalences are based on Model 1A (null model, individual as level 1 and 16 social strata as level 2). Reference categories: 50–59 years, female, had high education (tertiary/higher), Swedish-born. A black square indicates “Yes”, while a white square indicates “No”. For example, a stratum with all white squares consists of females aged 50–59 years who had high education and were Swedish-born; a stratum with all black squares consists of males aged 60–64 years who had low education and were foreign-born. Figures are sorted in ascending order based on the predictive prevalence of cardiometabolic multimorbidity

Figure 1 presents the Model 1A predicted prevalence of CMD status for 16 social strata based on I-MAIHDA. Overall, 60–64-year-old males across strata consistently showed a higher prevalence of any CMD and cardiometabolic multimorbidity. The lowest prevalence of cardiometabolic multimorbidity was observed among 50–59-year-old females with high education, irrespective of whether they were Swedish-born (0.8%, 95% CI 0.6–1.1) or foreign-born (0.9%, 95% CI 0.4–1.7). Conversely, strata consisting of 60–64-year-old males with low education, either foreign-born (10.0%, 95% CI 7.3–13.2) or Swedish-born (7.4%, 95% CI 6.4–8.4) had the highest prevalence of cardiometabolic multimorbidity.

The measure of GCE (Table 2, Model 1A) shows a moderate VPC of 6.4% for any CMD, indicating that only 6.4% of the total variance in the presence of any CMD was due to differences between social strata. In contrast, the VPC for cardiometabolic multimorbidity was substantially higher at 18.0%, suggesting that social strata play a substantial share in explaining the total individual variance of cardiometabolic multimorbidity. In other words, cardiometabolic multimorbidity clusters within social strata. The DA shows a poor AUC for any CMD (AUC = 0.61, 0.60–0.62) and a poor AUC for cardiometabolic multimorbidity (AUC = 0.69, 0.68–0.71).

**Table 2:** Results from the intersectional multilevel analysis of individual heterogeneity and discriminatory accuracy (I-MAIHDA)

Variables	Any CMD				Cardiometabolic multimorbidity			
	Model 1A	Model 1B	Model 2	Model 3	Model 1A	Model 1B	Model 2	Model 3
	Null model	Null model	Partially adjusted model	Intersectional interaction model	Null model	Null model	Partially adjusted model	Intersectional interaction model
**Measures of association (fixed effects)**								
Odds ratio (95% CI)								
Age (ref. 50–59 years)								
60–64 years			1.72 (1.49–1.98)	1.75 (1.65–1.87)			2.66 (2.00–3.55)	2.57 (2.21–2.98)
Sex (ref. Female)								
Male			1.45 (1.23–1.70)	1.48 (1.39–1.57)			2.47 (1.87–3.27)	2.39 (2.03–2.81)
Educational attainment (ref. Tertiary)								
Secondary/lower			1.53 (1.28–1.84)	1.49 (1.40–1.59)			1.93 (1.35–2.76)	1.79 (1.52–2.11)
Country of birth (ref. Swedish-born)								
Foreign-born			1.27 (1.05–1.55)	1.34 (1.24–1.44)			1.31 (0.94–1.83)	1.38 (1.15–1.67)
Area (ref. Gothenburg)								
Linköping			0.95 (0.70–1.29)	1.01 (0.91–1.13)			0.90 (0.44–1.82)	0.90 (0.69–1.18)
Malmö			0.96 (0.68–1.36)	1.02 (0.92–1.13)			1.30 (0.73–2.31)	1.30 (1.03–1.63)
Stockholm			1.29 (0.91–1.82)	1.38 (1.25–1.53)			0.90 (0.46–1.75)	0.99 (0.76–1.28)
Umeå			0.95 (0.67–1.35)	1.12 (0.99–1.28)			1.07 (0.52–2.21)	1.17 (0.88–1.57)
Uppsala			1.19 (0.86–1.65)	1.26 (1.14–1.40)			0.83 (0.42–1.64)	0.88 (0.67–1.14)
**Measures of variance (random effects)**								
Stratum-level variance (95% CI)	0.23 (0.10–0.49)	0.20 (0.14–0.28)	0.12 (0.08–0.17)^(a)^ 0.16 (0.11–0.22)^(b)^ 0.15 (0.11–0.22)^(c)^ 0.18 (0.13–0.26)^(d)^ 0.20 (0.14–0.28)^(e)^	0.00 (0.00–0.01)	0.75 (0.33–1.61)	0.59 (0.39–0.88)	0.35 (0.21–0.54)^(a)^ 0.38 (0.23–0.58) ^(b)^ 0.49 (0.31–0.74)^(c)^ 0.56 (0.36–0.85)^(d)^ 0.62 (0.39–0.94)^(e)^	0.01 (0.00–0.04)
VPC % (95% CI)	6.44 (3.07–12.90)	5.64 (4.06–7.73)	3.54 (2.45–4.99)^(a)^ 4.59 (3.23–6.37)^(b)^ 4.46 (3.15–6.23)^(c)^ 5.28 (3.78–7.32)^(d)^ 5.61 (3.99–7.77)^(e)^	0.11 (0.02–0.31)	18.04 (9.04–32.92)	15.05 (10.53–21.10)	9.69 (6.12–14.17)^(a)^ 10.24 (6.52–15.02)^(b)^ 12.86 (8.56–18.30)^(c)^ 14.56 (9.92–20.55)^(d)^ 15.71 (10.63–22.18)^(e)^	0.34 (0.02–1.35)
PCV	N/A	N/A	38.64%^(a)^ 19.39%^(b)^ 21.80%^(c)^ 6.60%^(d)^ 0.42%^(e)^	98.19%	N/A	N/A	39.44%^(a)^ 35.58%^(b)^ 16.71%^(c)^ 3.80%^(d)^ -5.21%^(e)^	98.07%
**Discriminatory accuracy**								
AUC (95% CI)	0.61 (0.60–0.62)	0.62 (0.62–0.63)	0.62 (0.62–0.63)	0.62 (0.62–0.63)	0.69 (0.68–0.71)	0.71 (0.70–0.73)	0.71 (0.70–0.73)	0.70 (0.69–0.72)

Notes: CMD: cardiometabolic disease, OR: odds ratio, CI: confidence interval

Model 1 A: Individuals (level 1) nested within 16 social strata (level 2); Model 1B: Individuals (level 1) nested within 96 socio-geographical strata (level 2)

Model 2: Individuals (level 1) nested within 96 socio-geographical strata (level 2), with *one variable* (a: age, b: sex, c: education, d: country of birth, e: areas) included at a time in the fixed effects

Model 3: Individuals (level 1) nested within 96 socio-geographical strata (level 2), with *all the five variables* included in the fixed effects

VPC = variance partition coefficient, indicates the percent of the total variation in the dependent variable that is attributable to the between–stratum level; PVC = proportional change in the variance, indicates the percent of the total between-stratum variance from the null model (Model 1B) that was explained after adjustment for additive main effects, AUC = area under the receiver operating characteristics curve, indicates the accuracy of social strata in discriminating individuals with cardiometabolic multimorbidity from those without cardiometabolic multimorbidity

**Fig. 2:**
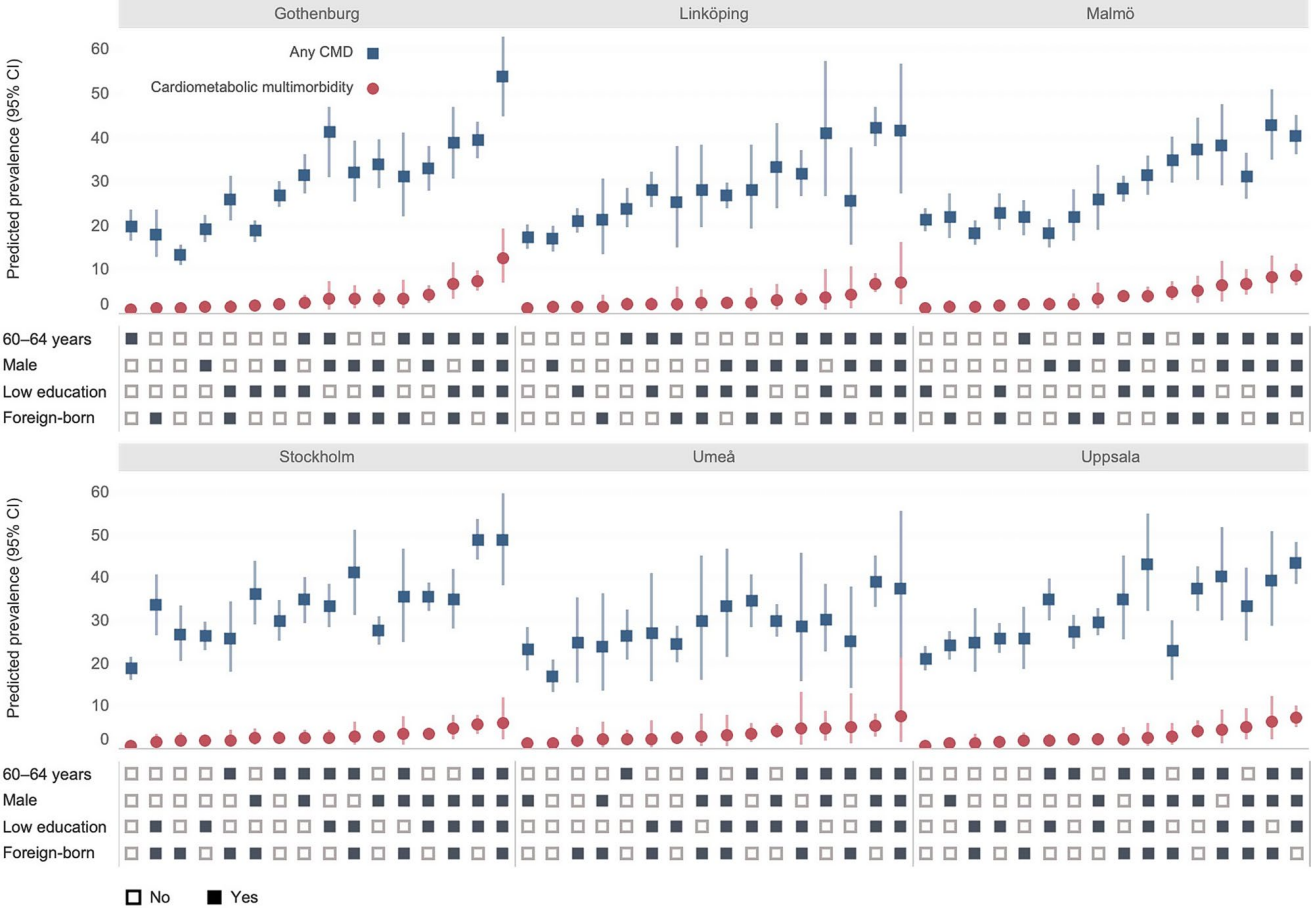
Predicted prevalence of any cardiometabolic disease and cardiometabolic multimorbidity by socio-geographical strata. Notes: CMD: Cardiometabolic disease; CI: Confidence interval. Predicted prevalences are based on Model 1B (null, individual as level 1 and 96 socio-geographical strata as level 2). A black square indicates “Yes”, while a white square indicates “No”. For example, a stratum with all white squares consists of females aged 50–59 years, with high education, and Swedish-born; a stratum with all black squares consists of males aged 60–64 years, with low education and foreign-born. Figures are sorted in ascending order based on the predictive prevalence of cardiometabolic multimorbidity

The predicted prevalence of CMD status across the 96 socio-geographical strata (Model 1B) is shown in Fig. 2 and Table S6 (Supplementary material 1). Across the six areas, the highest prevalence of cardiometabolic multimorbidity was observed in strata consisting of 60–64-year-old males with low education, with a mixed pattern in terms of country of birth. For example, 60–64-year-old males with low education and foreign-born reported the highest prevalence in Gothenburg (12.4%, 95% CI 7.1–19.3), Umeå (7.5%, 95% CI 1.6–21.3), Linköping (7.0%, 95% CI 2.0–16.3), and Stockholm (5.9%, 95% CI 2.2–12.0). In Malmö and Uppsala, the highest prevalence was found in 60–64-year-old Swedish-born males with low education (8.6%, 95% CI 6.3–11.3 in Malmö; 7.3%, 95% CI 5.0–10.0 in Uppsala). Across the six areas, the widest within-area disparity was observed in Gothenburg for both any CMD (53.8% in 60–64-year-old foreign-born male with low education vs. 13.3% in 50–59-year-old Swedish-born female with high education) and multimorbidity (12.4% for 60–64-year-old foreign-born males with low education vs. 1.0% for 60–64-year-old Swedish-born female with high education). The CIs were highly overlapped across the strata.

The VPCs obtained from Model 1B (Table 2) were 5.6% for any CMD and 15.0% for cardiometabolic multimorbidity, which were lower than those in Model 1A. The decrease in VPC, compared to Model 1A, suggests that adding six geographical areas to the model did not increase the total individual variance explained by intersectional strata for either outcome. However, the AUC for Model 1B was higher (AUC = 0.62, 95% CI 0.62–0.63 for any CMD and AUC = 0.71, 0.70–0.73 for cardiometabolic multimorbidity) compared to Model 1A, suggesting socio-geographical strata improved the model’s ability to discriminate between individuals with and without the outcome. Based on the AUC, socio-geographical strata were selected for further assessment in Models 2 and 3. The results for Model 2 and Model 3, using social strata, are presented in the Supplementary material 1 (Table S7). 

From the PCV in Model 2 (Table 2), age emerged as the most important variable in explaining the variance for any CMD, as indicated by the highest PCV. For example, after adjustment for age, the variance decreased from 0.20 (any CMD, Model 1B) to 0.12 (any CMD, Model 2(a)), resulting in a PCV of 38.6% for any CMD. This implies that 38.6% of the variance in any CMD in Model 1B was attributable to age. For cardiometabolic multimorbidity, a substantial change in variance was also observed in the age-adjusted model (Model 2(a), PCV = 39.4%), followed by the sex-adjusted model (Model 2(b), PCV = 35.6%).

**Fig. 3:**
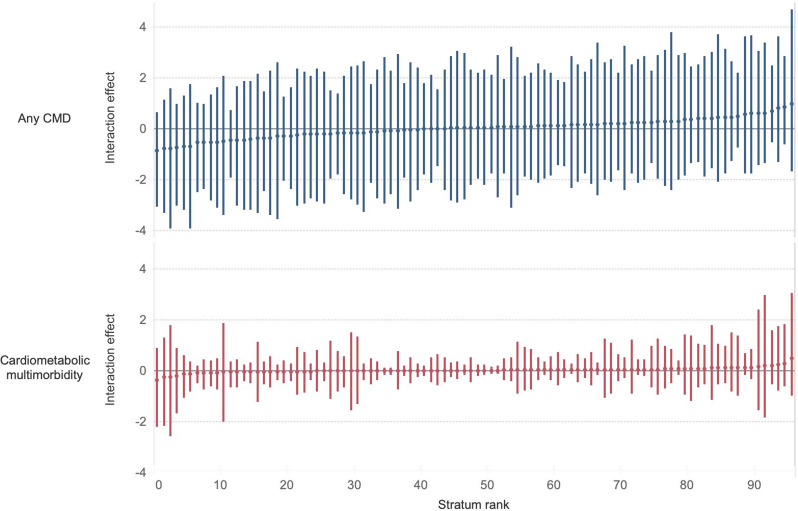
Intersectional interaction effects on the prevalence of any CMD and cardiometabolic multimorbidity based on Model 3, by socio-geographical strata. Notes: Interaction effect (*ARDI*_*j*_): the absolute risk due to the interaction effect (*u*_*j*_). Numbers and stratum ranks are available in Table S7

Figure 3 and Table S6 (Supplementary material 1) display the intersectional interaction effect (ARDI). The 95% CI of the ARDIs included the null for both outcomes, indicating no conclusive interaction effects. The VPC also dropped to 0.1% and 0.3% for any CMD and cardiometabolic multimorbidity, respectively (Table 2, Model 3). The small VPC in Model 3 implies that the additive effect of age, sex, education, country of birth, and areas, rather than multiplicative, explains most differences in the prevalence of any CMD and cardiometabolic multimorbidity across socio-geographical strata. Models 2 and 3 based on social strata (Supplementary material 1, Table S8 and Figure S2) also show consistent findings with no interactions.

The main effects ORs (Table 2, Model 3) also indicate that individuals aged 60–64 years (2.57, 95% CI 2.21–2.98), males (2.39, 95% CI 2.03–2.81), with low education (1.79, 95% 1.52–2.11), and who were foreign-born (1.38, 95% CI 1.15–1.67) had higher odds of having cardiometabolic multimorbidity, compared to those aged 50–59 years, females, had high education, and who were born in Sweden, respectively. There was generally no significant association observed between areas and cardiometabolic multimorbidity, except for Malmö (OR 1.30, 1.03–1.63).

### Sensitivity analyses

Our findings show a substantial drop in the prevalence of CMD when only self-reported information was used to define CMD. When relying solely on self-reports, the age- and sex-standardised prevalence decreased to 7.9% for any CMD and 0.6% for multimorbidity, compared with 24.4% and 2.8%, respectively, when combining multiple data sources (self-reports, register data, and glycaemic status assessments). Moreover, inconsistency in CMD classification between self-reported and multi-source information was greatest among foreign-born men aged 60–64 years with low education (Supplementary material 1, Table S9).

When diabetes and CVD were analysed separately, the vulnerability pattern remained: males aged 60–64 with low education, regardless of country of birth, had the highest predicted prevalence for both outcomes (Supplementary material 1, Table S10). Using employment status as a proxy for socioeconomic position revealed a similarly consistent pattern of vulnerability (Supplementary material 1, Table S11). With three categories of country of birth (Swedish-born, second-generation migrant, and foreign-born), foreign-born individuals generally had a higher prevalence of CMD and multimorbidity than their Swedish-born counterparts, while second-generation migrants often fell between the two strata (Supplementary material 1, Table S12). These results should be interpreted with caution due to small sample sizes in some strata.

## Discussion

This study is the first to apply intersectional theory and I-MAIHDA in mapping the disparities of cardiometabolic multimorbidity across sixteen social strata and six areas in Sweden. Three main findings were highlighted in the study. *First*, across the six areas, strata comprised of 60–64-year-old males with low education, irrespective of country of birth, had the highest prevalence of cardiometabolic multimorbidity. The highest prevalence was observed in 60–64-year-old foreign-born males with low education in Gothenburg and 60–64-year-old Swedish-born males with low education in Malmö. *Second*, based on the VPC, the GCE was moderate and high for any CMD and cardiometabolic multimorbidity, respectively. This implies that socio-geographical strata are highly relevant in explaining the disparities of cardiometabolic multimorbidity, but not for any CMD. The AUC shows a poor DA for any CMD and a low-end acceptable DA for cardiometabolic multimorbidity. *Third*, the intersectional interaction model discovered that the between-stratum differences were mainly explained by the additive main effect rather than the interaction effect (Model 3).

This study contributes to the emerging body of research supporting an intersectional approach over traditional epidemiological methods, stressing the importance of measuring DA in addition to the group averages [15, 19, 20, 33]. Despite being categorised based on similar characteristics, social groups are not homogeneous as they comprise multiple subpopulations with distinct cultural, historical and societal backgrounds [52]. Ignoring these social dynamics in health research may result in a lack of understanding of health disparities, which could result in misguided recommendations for reducing such disparities [53]. Thus, previous studies have advocated using an intersectional approach and DA analysis to move beyond a unidimensional understanding of health disparities to guide intervention planning in public health [15, 19, 33, 54].

Our findings show substantial differences in the prevalence of having any CMD between social/socio-geographical strata, with moderate VPC and low AUC. This corroborates the findings of Wemrell et al. (2017), where an intersectional approach was applied to investigate inequalities in ischaemic heart disease across social dimensions in Sweden [54]. They found significant differences in the average risk between social dimensions, as a combination of gender, income, time of living in Sweden, marital status and psychotropic medication, and ischaemic heart disease. However, similar to our finding, the measure of DA was also low. Thus, our study supports the argument that macro- and meso-level strategies are more relevant than interventions that exclusively target individuals within specific strata with high prevalence, to reduce disparities in CMD [19, 54].

Our findings also highlight a notable result for cardiometabolic multimorbidity: a high VPC of 15%, but a low-acceptable AUC of 0.71. A high VPC represents a strong *general contextual effect*, revealing that cardiometabolic multimorbidity is meaningfully structured by intersectional contexts. The VPC, which captures this contextual structuring, is not influenced by stratum size. In contrast, the AUC reflects *discriminatory accuracy*, that is, how well the intersectional strata discriminate individuals with and without the outcome. The AUC is sensitive to the distribution and size of the strata. Therefore, it is possible to observe a high VPC but a modest AUC: the contexts matter (large between-strata variance), yet most cases still occur among individuals in the more common “lower-risk” strata.

This apparent discrepancy reproduces Rose’s epidemiological paradox: most cases often arise from the group with the low average risk [55, 56]. Our data illustrates this perfectly. While low-educated, foreign-born males aged 60–64 years, particularly in Malmö and Gothenburg, had the highest predicted prevalence, low-educated Swedish-born males aged 50–59 years had much lower prevalence but accounted for many absolute cases due to their larger numbers (Supplementary material 1, Figure S3).

Given this situation, a high VPC but modest AUC indicates that interventions should not rely solely on identifying and stigmatising small, high-risk groups. Restricting interventions to only the most vulnerable strata would result in missed opportunities to intervene where most cases occur. Hence, these results suggest that the optimal prevention strategy is proportionate universalism, combining a universal backbone with intersection-specific reinforcement. In practical terms, this would involve implementing universal health dialogues (population-wide screening for blood pressure, glucose, BMI, and lifestyle counselling), while also applying targeted proportional intensification in vulnerable intersectional strata, such as workplace-based screening and culturally adapted support for older, low-educated, foreign-born men. Simultaneously, light-touch reinforcement, e.g., digital self-monitoring, could be used for groups with lower average risk but larger population size.

A 2025 systematic review found that the universal health dialogues in Sweden, such as the ‘Västerbotten Intervention Programme’ (VIP) in Västerbotten County and ‘Live for Life’ in Skaraborg County [57–59], were associated with reductions in CVD mortality and improvements in biological risk factors, but not in behavioural risk factors, except for healthy diet. The associations were also observed among individuals with lower socioeconomic status [59, 60]. However, a key limitation is that the inequality assessment was based on a single axis, treating the “low socioeconomic group” as homogeneous and thereby hiding within-group disparities [19, 59]. Thus, our intersectional analysis provides the evidence needed to refine targeted dialogues, ensuring that person-centred interventions can proactively reach vulnerable strata by considering their intersecting identities and specific needs, expectations, and health literacy [61].

A notable strength of this study is the application of I-MAIHDA using a high-quality population-based dataset. I-MAIHDA enhances our understanding of the social and geographical distribution of CMD multimorbidity to better inform more effective interventions and policies. I-MAIHDA allows the examination of social dynamics between multiple social dimensions in shaping disparities while equipping the information with DA to consider whether targeted intervention on specific vulnerable groups is more suitable over universal intervention from an epidemiological perspective. Additionally, incorporating geographical area into the model provides a more comprehensive assessment of whether area-specific interaction is required [47]. Furthermore, as MAIHDA requires high-quality datasets with sufficiently large sample sizes, the SCAPIS dataset, linked to register data, offers high-quality variables for assessing CMD multimorbidity disparities. Our findings indicated that relying exclusively on self-reported data likely underestimates both the prevalence and inequality of CMD, particularly among vulnerable groups who may experience greater barriers to accessing healthcare.

Our findings, however, are subject to several limitations. First, participation in SCAPIS was lower among individuals with lower socioeconomic status and non-Swedish-born than in the SCAPIS target population [62]. Since these groups typically have a higher disease burden, their underrepresentation likely led to an underestimation of the true magnitude of CMD disparities. This bias also reduced the precision of estimates in some intersectional strata, especially foreign-born living in Umeå, as reflected by wider 95% CIs. Furthermore, statistical adjustment using sample weights was not possible due to the unavailability of sample weights in the SCAPIS dataset. Nevertheless, the SCAPIS sample was reportedly similar to the target population in most of the baseline risk factors, which may partly mitigate concerns regarding the validity of the findings [62]. Second, to mitigate the potential reverse causation due to the cross-sectional nature of the dataset, some variables which are regarded as necessary in defining intersectional strata were not included in the model (e.g., employment status and income). Adding more dimension to construct the intersectional strata was also impossible due to the limitation of sample size (to ensure no stratum with *n* < 5). Third, the study could not consider information on CMD status at the primary healthcare level, as NPR only covers hospital-based visits, which could underestimate the prevalence reported. The measurement of the conditions was also retrospective since the register data linked to SCAPIS was only available in 2018. Future studies should measure the outcome prospectively. Fourth, constructing intersectional strata could be seen as oversimplifying social dimensions in the real social dynamics. For example, sex was solely assessed as binary (female/male), disregarding multiple gender identities, which has been reported to be one of the drivers of social disparities [6, 63]. Similarly, country of birth was classified simply as Swedish/foreign-born, which does not fully capture migration background, specific country origins, or the duration of residence in Sweden. However, constructing a more comprehensive intersectional strata necessitates a larger sample size, which our analysis could not accommodate. Finally, while I-MAIHDA is a powerful statistical tool, it does not directly address the root causes of health inequalities. Tackling these issues requires political will, structural interventions, and contextual understanding.

In conclusion, compared to the conventional unidimensional approach, I-MAIHDA provides a better mapping, highlighting variations in CMD disparities that are more/less pronounced in specific strata or areas. Our findings demonstrate a high VPC with a low-acceptable AUC for cardiometabolic multimorbidity. This evidence emphasises the importance of strengthening universal interventions for preventing cardiometabolic disease yet simultaneously tailoring the intervention to the characteristics of vulnerable social strata who already have or have a higher probability of having cardiometabolic multimorbidity.

## Supplementary Information

Below is the link to the electronic supplementary material.

Supplementary Material 1

## Data Availability

Data underlying this study cannot be shared publicly due to the privacy of individuals that participated in the study. However, all data used in this study are available from SCAPIS (www.scapis.org) for researcher, within applicable policies.
