# Supplementary material for: Socio-geographical disparities in cardiometabolic multimorbidity in Sweden: an Intersectional Multilevel Analysis of Individual Heterogeneity and Discriminatory Accuracy (I-MAIHDA)
Source: Int J Equity Health. 2025 Nov 4;24:301. doi: 10.1186/s12939-025-02684-z (PMC12584395; doi:10.1186/s12939-025-02684-z)
Supplement: Supplementary file 1 — Supplementary Material 1 [file 12939_2025_2684_MOESM1_ESM.docx]

# Appendices

Table S1. Number of observations with missing data

| Variable | Missing (n) | % |
| --- | --- | --- |
| **All SCAPIS respondents** | **30154** |  |
| Excluded respondents due to no consent for linking SCAPIS to register data | 124 |  |
| **Eligible respondents for analysis** | **30,030** | **100** |
| Variables with missing value | 937 | 3.1 |
| **Main independent variables** | | |
| Age | 0 | 0 |
| Sex | 0 | 0 |
| Education | 846 | 2.8 |
| Country of birth | 813 | 2.7 |
| Study sites | 0 | 0 |
| **N without missing value** | **29,093** | **96.9** |

**Notes:** Some respondents had multiple variables with missing values.

Table S2. ICD-10 and ATC codes for classification of cardiometabolic disease

| **Diagnoses** | **ICD-9 Codes** | **ICD-10 Codes** | **ATC Codes** |
| --- | --- | --- | --- |
| Type 2 diabetes | 250 | E11–E14 | A10A (for those without Type 1 diabetes), A10B |
| Heart disease |  |  |  |
| Coronary heart disease | 410–414 | I20–I25 |  |
| Heart failure | 428 | I50 |  |
| Stroke |  |  |  |
| Ischemic stroke | 433, 434 | I63 |  |
| Hemorrhagic stroke | 430–432 | I60–I62 |  |
| Unspecified stroke | 436, 437 | I64, I67, I68 |  |

**References:**

Dove A, Guo J, Marseglia A, Fastbom J, Vetrano DL, Fratiglioni L, Pedersen NL, Xu W. Cardiometabolic multimorbidity and incident dementia: the Swedish twin registry. Eur Heart J. 2023 Feb 14;44(7):573-582.

Canoy D, Tran J, Zottoli M, Ramakrishnan R, Hassaine A, Rao S, Li Y, Salimi-Khorshidi G, Norton R, Rahimi K. Association between cardiometabolic disease multimorbidity and all-cause mortality in 2 million women and men registered in UK general practices. BMC Med. 2021 Oct 28;19(1):258.

Table S3. Variables’ definition

| **Variables** | **Original variables: description** | **Variables' measurement** |
| --- | --- | --- |
| **Outcome** |  |  |
| Cardiometabolic disease | The assessment of cardiometabolic disease is based on self-reported information, national patient register (inpatients & outpatients), and drug register.  Type 2 diabetes:  Self-reported information  cqhe038: Diabetes, doctor-diagnosed, self-reported  Measurement  Diabetes: Classification of glycaemic status  according to baseline examination results, including elevated HbA1c, impaired fasting glucose and diabetes mellitus (fasting p-glucose ≥6,1 mmol/l and HbA1c > 42mmol)  Patient and drug register  ICD-10 Code: E11–14 (see S2 Table)  ATC Code: A10  Heart disease:  Self-reported information  cqhe002: Myocardial infarction, doctor-diagnosed, self-reported  cqhe007: Angina pectoris, doctor-diagnosed, self-reported  cqhe011: Heart failure, doctor-diagnosed, self-reported  cqhe017: CABG/PCI intervention  Patient register  ICD-10 Code: I20–25, I50 (see Table S2)  Stroke:  Self-reported information  cqhe029: Stroke, doctor-diagnosed, self-reported  Patient register  ICD-10 Code: I60–I64, 67–68 (see Table S2) | Type 2 diabetes: 0: No  1: Yes (had the condition based on self-reported data and/or measurement and/or register data)  Heart disease:  0: No  1: Yes (had the condition based on self-reported data and/or register data)  Stroke:  0: No  1: Yes (had the condition based on self-reported data and/or register data)  Cardiometabolic disease  0: No CMD  1: Single CMD (had 1 cardiometabolic disease)  2: Cardiometabolic multimorbidity (had ≥2 cardiometabolic diseases) |
| **Social dimensions** |  |  |
| Age | AgeAtVisitOne: Age at study visit 1, rounded to 1 decimal | 0: 50–59 years  1: 60–64 years |
| Sex | Sex | 0: Females  1: Males |
| Educational attainment | cqed001: Highest completed level of education | 0: High (at least university/college)  1: Low (high school/vocation school or lower) |
| Country of birth | cqli009: Born in Sweden (SCAPIS main participant)  cqli013: Country of birth, mother  cqli014: Country of birth, father | Main analysis:  0: Swedish-born (participant was born in Sweden)  1: Foreign-born (participant was born outside Sweden)  Sensitivity analysis:  0: Swedish-born (participant born outside or within Sweden with at least one Swedish-born parent)  1: Second-generation migrant (participant born in Sweden with both foreign-born parents)  1: Foreign-born (participant born outside Sweden with both foreign-born parents) |
| **Geographical area** |  |  |
| Area | Site: Site of examination | 0: Gothenburg  1: Linköping  2: Malmö  3: Stockholm  4: Umeå  5: Uppsala |

Table S4. Sample size for each social stratum

| **Social strata**  **(Age, sex, education, country of birth)** | **n** | **No CMD** | **Single CMD** | **Multimorbidity** |
| --- | --- | --- | --- | --- |
|  |  | % | % | % |
| 50–59 years Female Tertiary Swedish-born | 4139 | 83.0 | 16.3 | 0.8 |
| 50–59 years Female Tertiary Foreign-born | 759 | 78.7 | 20.7 | 0.7 |
| 50–59 years Female Secondary/lower Swedish-born | 4165 | 77.8 | 20.6 | 1.6 |
| 50–59 years Female Secondary/lower Foreign-born | 927 | 74.3 | 24.6 | 1.1 |
| 50–59 years Male Tertiary Swedish-born | 3209 | 78.9 | 19.5 | 1.6 |
| 50–59 years Male Tertiary Foreign-born | 631 | 71.6 | 25.8 | 2.5 |
| 50–59 years Male Secondary/lower Swedish-born | 4605 | 70.7 | 26.3 | 3.0 |
| 50–59 years Male Secondary/lower Foreign-born | 907 | 65.4 | 30.0 | 4.6 |
| 60–64 years Female Tertiary Swedish-born | 2133 | 75.9 | 22.4 | 1.8 |
| 60–64 years Female Tertiary Foreign-born | 379 | 72.3 | 24.8 | 2.9 |
| 60–64 years Female Secondary/lower Swedish-born | 2101 | 68.0 | 29.2 | 2.8 |
| 60–64 years Female Secondary/lower Foreign-born | 429 | 58.5 | 35.4 | 6.1 |
| 60–64 years Male Tertiary Swedish-born | 1594 | 66.2 | 29.2 | 4.6 |
| 60–64 years Male Tertiary Foreign-born | 289 | 60.6 | 33.2 | 6.2 |
| 60–64 years Male Secondary/lower Swedish-born | 2457 | 57.0 | 35.6 | 7.4 |
| 60–64 years Male Secondary/lower Foreign-born | 369 | 48.0 | 41.5 | 10.6 |

Notes: Row percentages

Table S5. Sample size for each socio-geographical stratum

| **Socio-geographical strata**  **(Area, age, sex, education, country of birth)** | **n** | **No CMD** | **Single CMD** | **Multimorbidity** |
| --- | --- | --- | --- | --- |
|  |  | n (%) | n (%) | n (%) |
| Göteborg 50–59 years Female Tertiary Swedish-born | 861 | 87.2 | 11.8 | 0.9 |
| Göteborg 50–59 years Female Tertiary Foreign-born | 165 | 84.2 | 15.8 | 0.0 |
| Göteborg 50–59 years Female Secondary/lower Swedish-born | 863 | 81.6 | 16.6 | 1.9 |
| Göteborg 50–59 years Female Secondary/lower Foreign-born | 264 | 74.2 | 24.6 | 1.1 |
| Göteborg 50–59 years Male Tertiary Swedish-born | 665 | 81.2 | 17.6 | 1.2 |
| Göteborg 50–59 years Male Tertiary Foreign-born | 145 | 67.6 | 29.0 | 3.4 |
| Göteborg 50–59 years Male Secondary/lower Swedish-born | 892 | 73.1 | 24.7 | 2.2 |
| Göteborg 50–59 years Male Secondary/lower Foreign-born | 253 | 65.6 | 30.8 | 3.6 |
| Göteborg 60–64 years Female Tertiary Swedish-born | 471 | 80.7 | 18.7 | 0.6 |
| Göteborg 60–64 years Female Tertiary Foreign-born | 73 | 68.5 | 27.4 | 4.1 |
| Göteborg 60–64 years Female Secondary/lower Swedish-born | 395 | 68.4 | 29.1 | 2.5 |
| Göteborg 60–64 years Female Secondary/lower Foreign-born | 121 | 59.5 | 32.2 | 8.3 |
| Göteborg 60–64 years Male Tertiary Swedish-born | 326 | 66.9 | 28.5 | 4.6 |
| Göteborg 60–64 years Male Tertiary Foreign-born | 54 | 53.7 | 42.6 | 3.7 |
| Göteborg 60–64 years Male Secondary/lower Swedish-born | 487 | 60.2 | 32.2 | 7.6 |
| Göteborg 60–64 years Male Secondary/lower Foreign-born | 98 | 40.8 | 43.9 | 15.3 |
| Linköping 50–59 years Female Tertiary Swedish-born | 691 | 83.1 | 16.1 | 0.9 |
| Linköping 50–59 years Female Tertiary Foreign-born | 62 | 82.3 | 17.7 | 0.0 |
| Linköping 50–59 years Female Secondary/lower Swedish-born | 812 | 79.3 | 19.2 | 1.5 |
| Linköping 50–59 years Female Secondary/lower Foreign-born | 65 | 72.3 | 26.2 | 1.5 |
| Linköping 50–59 years Male Tertiary Swedish-born | 588 | 83.5 | 15.3 | 1.2 |
| Linköping 50–59 years Male Tertiary Foreign-born | 66 | 65.2 | 31.8 | 3.0 |
| Linköping 50–59 years Male Secondary/lower Swedish-born | 903 | 73.4 | 24.3 | 2.3 |
| Linköping 50–59 years Male Secondary/lower Foreign-born | 58 | 72.4 | 25.9 | 1.7 |
| Linköping 60–64 years Female Tertiary Swedish-born | 329 | 76.6 | 21.3 | 2.1 |
| Linköping 60–64 years Female Tertiary Foreign-born | 24 | 79.2 | 20.8 | 0.0 |
| Linköping 60–64 years Female Secondary/lower Swedish-born | 460 | 72.0 | 25.9 | 2.2 |
| Linköping 60–64 years Female Secondary/lower Foreign-born | 18 | 44.4 | 50.0 | 5.6 |
| Linköping 60–64 years Male Tertiary Swedish-born | 289 | 67.8 | 28.7 | 3.5 |
| Linköping 60–64 years Male Tertiary Foreign-born | 31 | 77.4 | 16.1 | 6.5 |
| Linköping 60–64 years Male Secondary/lower Swedish-born | 485 | 57.1 | 35.7 | 7.2 |
| Linköping 60–64 years Male Secondary/lower Foreign-born | 20 | 45.0 | 40.0 | 15.0 |
| Malmö 50–59 years Female Tertiary Swedish-born | 725 | 82.2 | 16.4 | 1.4 |
| Malmö 50–59 years Female Tertiary Foreign-born | 218 | 78.9 | 20.2 | 0.9 |
| Malmö 50–59 years Female Secondary/lower Swedish-born | 858 | 79.0 | 19.8 | 1.2 |
| Malmö 50–59 years Female Secondary/lower Foreign-born | 343 | 77.6 | 21.0 | 1.5 |
| Malmö 50–59 years Male Tertiary Swedish-born | 502 | 82.5 | 15.3 | 2.2 |
| Malmö 50–59 years Male Tertiary Foreign-born | 152 | 79.6 | 18.4 | 2.0 |
| Malmö 50–59 years Male Secondary/lower Swedish-born | 815 | 71.8 | 24.3 | 3.9 |
| Malmö 50–59 years Male Secondary/lower Foreign-born | 316 | 64.6 | 30.1 | 5.4 |
| Malmö 60–64 years Female Tertiary Swedish-born | 376 | 78.7 | 19.4 | 1.9 |
| Malmö 60–64 years Female Tertiary Foreign-born | 103 | 74.8 | 21.4 | 3.9 |
| Malmö 60–64 years Female Secondary/lower Swedish-born | 414 | 68.6 | 27.1 | 4.3 |
| Malmö 60–64 years Female Secondary/lower Foreign-born | 153 | 61.4 | 32.7 | 5.9 |
| Malmö 60–64 years Male Tertiary Swedish-born | 283 | 68.9 | 23.7 | 7.4 |
| Malmö 60–64 years Male Tertiary Foreign-born | 84 | 59.5 | 32.1 | 8.3 |
| Malmö 60–64 years Male Secondary/lower Swedish-born | 483 | 59.0 | 31.9 | 9.1 |
| Malmö 60–64 years Male Secondary/lower Foreign-born | 129 | 55.0 | 34.9 | 10.1 |
| Stockholm 50–59 years Female Tertiary Swedish-born | 720 | 81.8 | 17.9 | 0.3 |
| Stockholm 50–59 years Female Tertiary Foreign-born | 159 | 73.6 | 25.2 | 1.3 |
| Stockholm 50–59 years Female Secondary/lower Swedish-born | 655 | 73.7 | 24.4 | 1.8 |
| Stockholm 50–59 years Female Secondary/lower Foreign-born | 137 | 65.7 | 33.6 | 0.7 |
| Stockholm 50–59 years Male Tertiary Swedish-born | 627 | 72.6 | 24.6 | 2.9 |
| Stockholm 50–59 years Male Tertiary Foreign-born | 137 | 62.8 | 35.0 | 2.2 |
| Stockholm 50–59 years Male Secondary/lower Swedish-born | 779 | 64.3 | 32.1 | 3.6 |
| Stockholm 50–59 years Male Secondary/lower Foreign-born | 154 | 64.3 | 30.5 | 5.2 |
| Stockholm 60–64 years Female Tertiary Swedish-born | 337 | 70.0 | 27.6 | 2.4 |
| Stockholm 60–64 years Female Tertiary Foreign-born | 90 | 75.6 | 23.3 | 1.1 |
| Stockholm 60–64 years Female Secondary/lower Swedish-born | 309 | 66.3 | 31.1 | 2.6 |
| Stockholm 60–64 years Female Secondary/lower Foreign-born | 76 | 55.3 | 42.1 | 2.6 |
| Stockholm 60–64 years Male Tertiary Swedish-born | 278 | 64.7 | 32.7 | 2.5 |
| Stockholm 60–64 years Male Tertiary Foreign-born | 52 | 61.5 | 34.6 | 3.8 |
| Stockholm 60–64 years Male Secondary/lower Swedish-born | 388 | 50.0 | 44.1 | 5.9 |
| Stockholm 60–64 years Male Secondary/lower Foreign-born | 63 | 44.4 | 47.6 | 7.9 |
| Umeå 50–59 years Female Tertiary Swedish-born | 379 | 84.2 | 14.8 | 1.1 |
| Umeå 50–59 years Female Tertiary Foreign-born | 42 | 78.6 | 21.4 | 0.0 |
| Umeå 50–59 years Female Secondary/lower Swedish-born | 381 | 75.9 | 21.8 | 2.4 |
| Umeå 50–59 years Female Secondary/lower Foreign-born | 21 | 76.2 | 23.8 | 0.0 |
| Umeå 50–59 years Male Tertiary Swedish-born | 252 | 77.4 | 21.8 | 0.8 |
| Umeå 50–59 years Male Tertiary Foreign-born | 27 | 81.5 | 18.5 | 0.0 |
| Umeå 50–59 years Male Secondary/lower Swedish-born | 500 | 70.2 | 25.6 | 4.2 |
| Umeå 50–59 years Male Secondary/lower Foreign-born | 30 | 63.3 | 33.3 | 3.3 |
| Umeå 60–64 years Female Tertiary Swedish-born | 185 | 74.1 | 23.8 | 2.2 |
| Umeå 60–64 years Female Tertiary Foreign-born | 21 | 81.0 | 9.5 | 9.5 |
| Umeå 60–64 years Female Secondary/lower Swedish-born | 196 | 64.8 | 31.6 | 3.6 |
| Umeå 60–64 years Female Secondary/lower Foreign-born | 8 | 75.0 | 12.5 | 12.5 |
| Umeå 60–64 years Male Tertiary Swedish-born | 108 | 69.4 | 25.0 | 5.6 |
| Umeå 60–64 years Male Tertiary Foreign-born | 10 | 70.0 | 30.0 | 0.0 |
| Umeå 60–64 years Male Secondary/lower Swedish-born | 227 | 59.9 | 34.4 | 5.7 |
| Umeå 60–64 years Male Secondary/lower Foreign-born | 6 | 33.3 | 33.3 | 33.3 |
| Uppsala 50–59 years Female Tertiary Swedish-born | 763 | 79.3 | 20.4 | 0.3 |
| Uppsala 50–59 years Female Tertiary Foreign-born | 113 | 75.2 | 23.9 | 0.9 |
| Uppsala 50–59 years Female Secondary/lower Swedish-born | 596 | 74.3 | 24.2 | 1.5 |
| Uppsala 50–59 years Female Secondary/lower Foreign-born | 97 | 76.3 | 23.7 | 0.0 |
| Uppsala 50–59 years Male Tertiary Swedish-born | 575 | 76.2 | 23.0 | 0.9 |
| Uppsala 50–59 years Male Tertiary Foreign-born | 104 | 78.8 | 18.3 | 2.9 |
| Uppsala 50–59 years Male Secondary/lower Swedish-born | 716 | 70.5 | 27.4 | 2.1 |
| Uppsala 50–59 years Male Secondary/lower Foreign-born | 96 | 65.6 | 28.1 | 6.2 |
| Uppsala 60–64 years Female Tertiary Swedish-born | 435 | 72.9 | 25.1 | 2.1 |
| Uppsala 60–64 years Female Tertiary Foreign-born | 68 | 63.2 | 35.3 | 1.5 |
| Uppsala 60–64 years Female Secondary/lower Swedish-born | 327 | 64.8 | 33.3 | 1.8 |
| Uppsala 60–64 years Female Secondary/lower Foreign-born | 53 | 54.7 | 39.6 | 5.7 |
| Uppsala 60–64 years Male Tertiary Swedish-born | 310 | 61.9 | 33.5 | 4.5 |
| Uppsala 60–64 years Male Tertiary Foreign-born | 58 | 56.9 | 34.5 | 8.6 |
| Uppsala 60–64 years Male Secondary/lower Swedish-born | 387 | 55.8 | 36.4 | 7.8 |
| Uppsala 60–64 years Male Secondary/lower Foreign-born | 53 | 50.9 | 47.2 | 1.9 |
| Notes: Row percentages |  |  |  |  |

Table S6. The predicted prevalences of any CMD and cardiometabolic multimorbidity based on main effect and interaction effects, sorted by socio-geographical strata (stratum ranks are sorted in ascending order by Model 3, interaction effect)

| **Outcome** | **Rank** | **Socio-geographical strata**  **(Area, age, sex, education, country of birth)** | **Model 1** | **Model 3 (main effect+interaction)** | **Model 3 (main effect)** | **Model 3 (interaction)** |
| --- | --- | --- | --- | --- | --- | --- |
| Any CMD | 1 | Linköping 50–59 years Male Tertiary Swedish-born | 17.1 (14.2–20.1) | 19.4 (17.0–21.3) | 20.2 (18.7–21.8) | -0.8 (-3.0–0.7) |
| Any CMD | 2 | Uppsala 60–64 years Female Tertiary Foreign-born | 29.5 (26.4–32.7) | 31.2 (28.7–33.6) | 32.0 (30.1–34.0) | -0.8 (-3.3–1.1) |
| Any CMD | 3 | Stockholm 60–64 years Female Secondary/lower Foreign-born | 33.4 (28.3–38.7) | 37.2 (33.9–40.1) | 38.0 (35.8–40.2) | -0.8 (-3.9–1.6) |
| Any CMD | 4 | Göteborg 60–64 years Female Tertiary Swedish-born | 19.9 (16.5–23.5) | 22.1 (19.8–24.3) | 22.9 (21.3–24.6) | -0.7 (-3.0–1.0) |
| Any CMD | 5 | Linköping 60–64 years Female Secondary/lower Swedish-born | 28.1 (24.3–32.1) | 30.2 (27.5–32.8) | 31.0 (28.9–33.1) | -0.7 (-3.2–1.3) |
| Any CMD | 6 | Stockholm 60–64 years Female Secondary/lower Swedish-born | 25.6 (17.9–34.4) | 34.7 (31.0–38.1) | 35.4 (32.9–38.0) | -0.7 (-3.9–1.7) |
| Any CMD | 7 | Malmö 50–59 years Male Tertiary Swedish-born | 18.2 (15.1–21.4) | 19.8 (17.6–21.8) | 20.4 (18.9–21.9) | -0.6 (-2.5–1.0) |
| Any CMD | 8 | Göteborg 50–59 years Female Secondary/lower Swedish-born | 18.7 (16.3–21.3) | 19.6 (17.8–21.4) | 20.2 (18.8–21.7) | -0.5 (-2.4–1.0) |
| Any CMD | 9 | Malmö 50–59 years Female Secondary/lower Swedish-born | 22.9 (18.9–27.4) | 25.1 (22.5–27.5) | 25.7 (23.9–27.5) | -0.5 (-2.8–1.3) |
| Any CMD | 10 | Uppsala 60–64 years Female Tertiary Swedish-born | 22.8 (16.1–29.9) | 29.1 (25.8–32.2) | 29.6 (27.4–31.9) | -0.5 (-3.1–1.6) |
| Any CMD | 11 | Stockholm 50–59 years Male Tertiary Foreign-born | 34.8 (28.0–42.1) | 40.3 (36.8–43.6) | 40.8 (38.4–43.4) | -0.5 (-3.4–2.1) |
| Any CMD | 12 | Göteborg 50–59 years Female Tertiary Swedish-born | 13.3 (11.2–15.6) | 14.0 (12.6–15.4) | 14.5 (13.3–15.7) | -0.4 (-1.9–0.7) |
| Any CMD | 13 | Malmö 60–64 years Female Tertiary Swedish-born | 21.9 (16.5–28.2) | 25.0 (22.0–27.7) | 25.5 (23.5–27.4) | -0.4 (-3.0–1.7) |
| Any CMD | 14 | Stockholm 60–64 years Male Tertiary Swedish-born | 34.8 (29.4–40.2) | 37.3 (34.2–40.2) | 37.7 (35.5–39.9) | -0.4 (-3.2–1.9) |
| Any CMD | 15 | Uppsala 50–59 years Female Secondary/lower Swedish-born | 24.9 (17.8–32.9) | 29.5 (26.1–32.6) | 29.9 (27.7–32.2) | -0.4 (-3.2–1.9) |
| Any CMD | 16 | Umeå 60–64 years Male Secondary/lower Swedish-born | 39.0 (33.1–45.0) | 42.0 (38.6–45.4) | 42.4 (39.7–45.2) | -0.4 (-3.3–2.2) |
| Any CMD | 17 | Malmö 50–59 years Male Secondary/lower Swedish-born | 21.8 (17.9–25.8) | 22.9 (20.5–25.1) | 23.3 (21.7–25.0) | -0.4 (-2.5–1.4) |
| Any CMD | 18 | Linköping 60–64 years Male Tertiary Foreign-born | 25.6 (15.7–37.7) | 36.9 (32.9–40.6) | 37.2 (34.4–40.0) | -0.4 (-3.4–2.3) |
| Any CMD | 19 | Stockholm 60–64 years Male Tertiary Foreign-born | 35.5 (24.9–46.8) | 44.4 (40.3–48.2) | 44.7 (42.1–47.6) | -0.3 (-3.6–2.6) |
| Any CMD | 20 | Göteborg 50–59 years Male Tertiary Swedish-born | 19.2 (16.3–22.3) | 19.7 (17.8–21.6) | 20.0 (18.5–21.5) | -0.3 (-2.0–1.3) |
| Any CMD | 21 | Linköping 50–59 years Male Secondary/lower Swedish-born | 26.7 (24.0–29.6) | 27.1 (25.0–29.2) | 27.4 (25.6–29.3) | -0.3 (-2.4–1.6) |
| Any CMD | 22 | Uppsala 50–59 years Male Tertiary Foreign-born | 33.4 (25.3–42.2) | 38.3 (34.8–41.8) | 38.6 (36.2–41.1) | -0.3 (-3.0–2.3) |
| Any CMD | 23 | Uppsala 60–64 years Male Secondary/lower Swedish-born | 43.4 (38.6–48.2) | 45.0 (42.0–47.8) | 45.2 (42.9–47.5) | -0.2 (-2.9–2.2) |
| Any CMD | 24 | Malmö 60–64 years Female Secondary/lower Swedish-born | 26.1 (19.1–33.9) | 28.6 (25.5–31.7) | 28.8 (26.7–31.0) | -0.2 (-2.7–2.1) |
| Any CMD | 25 | Linköping 50–59 years Male Secondary/lower Foreign-born | 28.2 (19.4–38.4) | 33.3 (29.8–36.9) | 33.5 (31.1–36.1) | -0.2 (-2.8–2.4) |
| Any CMD | 26 | Umeå 60–64 years Male Tertiary Swedish-born | 30.3 (22.6–38.5) | 32.8 (29.4–36.3) | 33.0 (30.5–35.6) | -0.2 (-2.9–2.2) |
| Any CMD | 27 | Göteborg 50–59 years Female Tertiary Foreign-born | 17.8 (12.9–23.7) | 18.3 (16.1–20.5) | 18.5 (16.9–20.1) | -0.2 (-2.0–1.5) |
| Any CMD | 28 | Stockholm 50–59 years Female Tertiary Swedish-born | 18.6 (16.0–21.5) | 18.8 (17.0–20.6) | 19.0 (17.6–20.3) | -0.2 (-1.8–1.4) |
| Any CMD | 29 | Umeå 60–64 years Female Tertiary Swedish-born | 23.9 (13.6–36.3) | 27.1 (23.6–30.7) | 27.3 (24.7–30.1) | -0.2 (-2.6–2.1) |
| Any CMD | 30 | Umeå 60–64 years Female Secondary/lower Swedish-born | 24.9 (14.3–37.9) | 30.7 (26.9–34.5) | 30.9 (27.9–33.9) | -0.2 (-2.8–2.4) |
| Any CMD | 31 | Malmö 60–64 years Male Secondary/lower Foreign-born | 42.7 (35.0–50.8) | 47.0 (43.4–50.6) | 47.2 (44.6–49.7) | -0.2 (-3.0–2.5) |
| Any CMD | 32 | Uppsala 60–64 years Male Secondary/lower Foreign-born | 43.2 (32.2–54.9) | 52.3 (48.3–56.1) | 52.4 (49.6–55.3) | -0.1 (-3.3–2.6) |
| Any CMD | 33 | Göteborg 50–59 years Male Secondary/lower Swedish-born | 27.0 (24.2–29.9) | 27.1 (25.1–29.1) | 27.2 (25.5–28.9) | -0.1 (-2.1–1.7) |
| Any CMD | 34 | Uppsala 60–64 years Female Secondary/lower Foreign-born | 34.8 (30.0–39.8) | 35.7 (32.7–38.6) | 35.8 (33.7–38.1) | -0.1 (-2.7–2.3) |
| Any CMD | 35 | Umeå 50–59 years Male Secondary/lower Foreign-born | 28.7 (15.6–45.6) | 39.9 (35.6–44.2) | 40.0 (36.7–43.3) | -0.1 (-2.9–2.8) |
| Any CMD | 36 | Linköping 60–64 years Female Tertiary Foreign-born | 25.4 (15.1–38.1) | 28.6 (25.3–31.9) | 28.6 (26.3–31.1) | -0.1 (-2.6–2.3) |
| Any CMD | 37 | Umeå 60–64 years Male Tertiary Foreign-born | 29.7 (16.0–45.0) | 39.6 (35.2–44.1) | 39.7 (36.4–43.1) | -0.1 (-3.1–2.9) |
| Any CMD | 38 | Uppsala 50–59 years Male Tertiary Swedish-born | 24.1 (20.8–27.5) | 23.9 (21.8–26.1) | 24.0 (22.3–25.7) | -0.1 (-1.9–1.8) |
| Any CMD | 39 | Umeå 50–59 years Male Tertiary Foreign-born | 33.3 (21.6–46.8) | 35.9 (31.9–40.0) | 35.9 (33.0–39.0) | -0.1 (-2.8–2.6) |
| Any CMD | 40 | Umeå 50–59 years Female Secondary/lower Swedish-born | 26.9 (15.7–40.9) | 27.5 (24.1–31.1) | 27.5 (25.0–30.3) | 0.0 (-2.4–2.4) |
| Any CMD | 41 | Linköping 50–59 years Female Tertiary Foreign-born | 21.4 (13.6–30.5) | 18.6 (16.2–21.1) | 18.6 (16.9–20.4) | 0.0 (-1.8–1.8) |
| Any CMD | 42 | Uppsala 50–59 years Male Secondary/lower Swedish-born | 27.2 (23.3–31.2) | 27.2 (24.9–29.7) | 27.2 (25.4–29.1) | 0.0 (-2.1–2.1) |
| Any CMD | 43 | Umeå 50–59 years Female Tertiary Swedish-born | 16.9 (13.4–20.7) | 16.0 (14.1–18.1) | 16.0 (14.5–17.7) | 0.0 (-1.5–1.5) |
| Any CMD | 44 | Göteborg 60–64 years Male Secondary/lower Swedish-born | 39.4 (35.2–43.6) | 39.6 (36.8–42.3) | 39.5 (37.4–41.7) | 0.0 (-2.4–2.3) |
| Any CMD | 45 | Stockholm 60–64 years Male Secondary/lower Foreign-born | 48.9 (38.3–59.8) | 54.7 (50.9–58.5) | 54.7 (52.0–57.5) | 0.0 (-2.8–2.9) |
| Any CMD | 46 | Uppsala 60–64 years Male Tertiary Foreign-born | 39.2 (28.6–50.7) | 42.5 (38.7–46.4) | 42.5 (39.7–45.3) | 0.0 (-2.9–3.1) |
| Any CMD | 47 | Stockholm 50–59 years Male Secondary/lower Foreign-born | 41.1 (31.1–51.3) | 45.0 (41.2–48.7) | 45.0 (42.2–47.8) | 0.0 (-2.8–3.0) |
| Any CMD | 48 | Malmö 60–64 years Male Tertiary Swedish-born | 31.0 (26.0–36.4) | 31.0 (28.3–33.8) | 30.9 (29.0–33.0) | 0.0 (-2.2–2.3) |
| Any CMD | 49 | Göteborg 50–59 years Female Secondary/lower Foreign-born | 26.0 (21.2–31.1) | 25.3 (22.7–28.0) | 25.3 (23.4–27.3) | 0.0 (-2.0–2.2) |
| Any CMD | 50 | Malmö 60–64 years Female Secondary/lower Foreign-born | 31.3 (27.0–35.9) | 31.2 (28.6–33.8) | 31.2 (29.2–33.2) | 0.0 (-2.2–2.3) |
| Any CMD | 51 | Umeå 50–59 years Female Tertiary Foreign-born | 24.7 (15.4–35.5) | 20.4 (17.5–23.3) | 20.3 (18.1–22.6) | 0.1 (-1.7–2.1) |
| Any CMD | 52 | Uppsala 50–59 years Male Secondary/lower Foreign-born | 40.3 (29.9–51.8) | 42.8 (39.0–46.6) | 42.7 (40.0–45.5) | 0.1 (-2.7–2.9) |
| Any CMD | 53 | Linköping 60–64 years Female Tertiary Swedish-born | 23.9 (19.6–28.6) | 23.2 (20.9–25.5) | 23.1 (21.4–24.8) | 0.1 (-1.8–2.0) |
| Any CMD | 54 | Umeå 60–64 years Male Secondary/lower Foreign-born | 37.3 (21.4–55.7) | 49.6 (45.0–54.2) | 49.6 (46.3–53.0) | 0.1 (-3.1–3.2) |
| Any CMD | 55 | Malmö 50–59 years Male Secondary/lower Foreign-born | 37.4 (30.3–44.4) | 37.8 (34.4–41.1) | 37.7 (35.3–40.1) | 0.1 (-2.6–2.8) |
| Any CMD | 56 | Umeå 50–59 years Male Tertiary Swedish-born | 23.2 (18.2–28.5) | 22.0 (19.6–24.7) | 22.0 (20.0–24.0) | 0.1 (-1.9–2.1) |
| Any CMD | 57 | Umeå 60–64 years Female Tertiary Foreign-born | 29.8 (26.1–33.8) | 29.7 (27.1–32.3) | 29.6 (27.3–31.8) | 0.1 (-2.0–2.2) |
| Any CMD | 58 | Linköping 50–59 years Female Secondary/lower Foreign-born | 28.1 (19.5–38.4) | 25.6 (22.6–28.7) | 25.5 (23.4–27.7) | 0.1 (-2.1–2.6) |
| Any CMD | 59 | Umeå 50–59 years Male Secondary/lower Swedish-born | 26.4 (20.8–32.5) | 25.1 (22.5–28.0) | 25.0 (22.9–27.3) | 0.1 (-2.0–2.3) |
| Any CMD | 60 | Stockholm 50–59 years Female Secondary/lower Foreign-born | 26.4 (23.1–29.8) | 26.0 (23.9–28.2) | 25.9 (24.2–27.6) | 0.1 (-1.8–2.2) |
| Any CMD | 61 | Linköping 50–59 years Female Secondary/lower Swedish-born | 21.0 (18.4–23.8) | 20.5 (18.7–22.4) | 20.4 (18.8–21.9) | 0.1 (-1.4–1.9) |
| Any CMD | 62 | Malmö 50–59 years Female Secondary/lower Foreign-born | 21.2 (18.7–23.9) | 20.7 (18.9–22.5) | 20.5 (19.1–22.0) | 0.1 (-1.5–1.8) |
| Any CMD | 63 | Uppsala 60–64 years Female Secondary/lower Swedish-born | 35.0 (25.6–45.1) | 33.5 (30.1–37.0) | 33.3 (30.9–35.9) | 0.1 (-2.3–2.8) |
| Any CMD | 64 | Stockholm 50–59 years Male Secondary/lower Swedish-born | 29.9 (25.3–34.8) | 29.3 (26.6–31.9) | 29.1 (27.2–31.0) | 0.2 (-2.1–2.5) |
| Any CMD | 65 | Uppsala 50–59 years Female Tertiary Foreign-born | 25.6 (18.6–33.0) | 22.4 (19.9–25.0) | 22.2 (20.4–24.1) | 0.2 (-1.8–2.2) |
| Any CMD | 66 | Göteborg 50–59 years Male Secondary/lower Foreign-born | 34.0 (28.4–39.7) | 33.5 (30.6–36.7) | 33.3 (31.1–35.6) | 0.2 (-2.2–2.7) |
| Any CMD | 67 | Linköping 60–64 years Male Secondary/lower Foreign-born | 41.5 (27.2–56.7) | 47.1 (43.1–51.4) | 46.9 (44.1–49.9) | 0.2 (-2.6–3.4) |
| Any CMD | 68 | Göteborg 60–64 years Female Secondary/lower Swedish-born | 31.5 (27.2–36.2) | 30.9 (28.4–33.6) | 30.7 (28.9–32.8) | 0.2 (-2.0–2.6) |
| Any CMD | 69 | Göteborg 60–64 years Female Tertiary Foreign-born | 31.0 (22.0–41.2) | 28.6 (25.5–31.9) | 28.4 (26.2–30.7) | 0.2 (-2.1–2.7) |
| Any CMD | 70 | Malmö 60–64 years Female Tertiary Foreign-born | 28.3 (25.3–31.4) | 27.8 (25.8–30.0) | 27.6 (25.9–29.4) | 0.2 (-1.6–2.2) |
| Any CMD | 71 | Malmö 60–64 years Male Tertiary Foreign-born | 38.1 (29.3–47.6) | 37.7 (34.3–41.3) | 37.4 (35.0–39.9) | 0.2 (-2.4–3.2) |
| Any CMD | 72 | Stockholm 50–59 years Female Tertiary Foreign-born | 26.8 (20.6–33.6) | 24.1 (21.5–26.9) | 23.8 (21.9–25.8) | 0.2 (-1.8–2.5) |
| Any CMD | 73 | Malmö 60–64 years Male Secondary/lower Swedish-born | 40.4 (36.2–44.9) | 40.3 (37.6–43.1) | 40.1 (37.9–42.3) | 0.2 (-2.1–2.7) |
| Any CMD | 74 | Stockholm 50–59 years Female Secondary/lower Swedish-born | 33.5 (26.4–40.8) | 32.1 (29.2–35.3) | 31.8 (29.5–34.1) | 0.2 (-2.0–2.8) |
| Any CMD | 75 | Malmö 50–59 years Female Tertiary Foreign-born | 22.0 (17.1–27.3) | 19.0 (16.9–21.3) | 18.8 (17.2–20.3) | 0.3 (-1.4–2.3) |
| Any CMD | 76 | Linköping 60–64 years Male Tertiary Swedish-born | 31.9 (26.7–37.1) | 31.0 (28.2–34.1) | 30.7 (28.7–32.8) | 0.3 (-1.9–2.9) |
| Any CMD | 77 | Göteborg 60–64 years Female Secondary/lower Foreign-born | 38.7 (30.5–46.9) | 37.5 (34.2–41.1) | 37.2 (34.8–39.7) | 0.3 (-2.2–3.1) |
| Any CMD | 78 | Linköping 60–64 years Female Secondary/lower Foreign-born | 41.1 (26.6–57.4) | 37.8 (33.9–42.1) | 37.5 (34.8–40.2) | 0.3 (-2.4–3.8) |
| Any CMD | 79 | Umeå 60–64 years Female Secondary/lower Foreign-born | 34.4 (28.3–40.7) | 33.6 (30.4–37.0) | 33.3 (30.8–35.8) | 0.3 (-2.0–2.9) |
| Any CMD | 80 | Malmö 50–59 years Male Tertiary Foreign-born | 34.9 (29.7–40.0) | 34.1 (31.4–37.1) | 33.8 (31.6–35.9) | 0.3 (-1.9–3.0) |
| Any CMD | 81 | Uppsala 50–59 years Female Secondary/lower Foreign-born | 25.9 (22.5–29.4) | 24.5 (22.4–26.9) | 24.2 (22.5–25.9) | 0.4 (-1.4–2.4) |
| Any CMD | 82 | Umeå 50–59 years Female Secondary/lower Foreign-born | 24.4 (20.3–28.8) | 22.5 (20.3–25.0) | 22.1 (20.3–24.1) | 0.4 (-1.3–2.5) |
| Any CMD | 83 | Göteborg 60–64 years Male Tertiary Swedish-born | 32.9 (27.9–38.1) | 30.9 (28.3–33.8) | 30.5 (28.4–32.5) | 0.4 (-1.9–2.8) |
| Any CMD | 84 | Linköping 50–59 years Male Tertiary Foreign-born | 33.3 (23.9–43.2) | 25.7 (22.9–28.9) | 25.3 (23.1–27.5) | 0.4 (-1.6–3.0) |
| Any CMD | 85 | Göteborg 60–64 years Male Tertiary Foreign-born | 41.3 (30.9–52.5) | 37.4 (33.8–41.4) | 36.9 (34.3–39.6) | 0.4 (-2.1–3.7) |
| Any CMD | 86 | Uppsala 60–64 years Male Tertiary Swedish-born | 37.4 (32.2–42.6) | 36.1 (33.3–39.1) | 35.6 (33.4–37.7) | 0.5 (-1.7–3.1) |
| Any CMD | 87 | Stockholm 50–59 years Male Tertiary Swedish-born | 27.5 (24.2–31.0) | 26.2 (24.1–28.4) | 25.7 (24.0–27.4) | 0.5 (-1.3–2.6) |
| Any CMD | 88 | Linköping 50–59 years Female Tertiary Swedish-born | 17.4 (14.7–20.2) | 15.1 (13.6–16.9) | 14.6 (13.4–15.8) | 0.5 (-0.7–2.2) |
| Any CMD | 89 | Stockholm 60–64 years Female Tertiary Swedish-born | 36.2 (29.1–43.7) | 32.2 (29.1–35.7) | 31.6 (29.4–34.0) | 0.6 (-1.8–3.6) |
| Any CMD | 90 | Stockholm 60–64 years Male Secondary/lower Swedish-born | 48.9 (44.1–53.8) | 48.1 (45.1–51.4) | 47.5 (45.1–49.8) | 0.6 (-1.8–3.6) |
| Any CMD | 91 | Stockholm 60–64 years Female Tertiary Foreign-born | 35.4 (32.2–38.7) | 34.6 (32.4–37.1) | 34.0 (32.1–36.0) | 0.6 (-1.4–3.0) |
| Any CMD | 92 | Göteborg 50–59 years Male Tertiary Foreign-born | 32.0 (25.4–39.4) | 25.7 (22.9–29.0) | 25.1 (23.0–27.1) | 0.6 (-1.3–3.4) |
| Any CMD | 93 | Malmö 50–59 years Female Tertiary Swedish-born | 18.3 (15.6–21.2) | 15.4 (13.9–17.3) | 14.8 (13.6–15.9) | 0.7 (-0.5–2.5) |
| Any CMD | 94 | Linköping 60–64 years Male Secondary/lower Swedish-born | 42.3 (38.0–46.9) | 40.6 (38.0–43.7) | 39.8 (37.6–42.2) | 0.8 (-1.3–3.6) |
| Any CMD | 95 | Uppsala 50–59 years Female Tertiary Swedish-born | 21.1 (18.3–23.9) | 18.4 (16.7–20.6) | 17.6 (16.2–18.9) | 0.8 (-0.6–2.8) |
| Any CMD | 96 | Göteborg 60–64 years Male Secondary/lower Foreign-born | 53.8 (44.8–62.8) | 47.6 (44.0–52.2) | 46.6 (44.0–49.4) | 1.0 (-1.7–4.7) |
| Multimorbidity | 1 | Umeå 60–64 years Male Secondary/lower Swedish-born | 5.2 (2.9–8.3) | 8.1 (6.0–10.3) | 8.4 (6.6–10.6) | -0.4 (-2.2–0.9) |
| Multimorbidity | 2 | Uppsala 60–64 years Male Secondary/lower Foreign-born | 2.5 (0.7–6.1) | 8.4 (5.8–11.2) | 8.7 (6.6–11.0) | -0.2 (-2.2–1.3) |
| Multimorbidity | 3 | Malmö 60–64 years Male Secondary/lower Foreign-born | 8.4 (4.6–13.1) | 12.0 (9.3–15.0) | 12.3 (10.0–14.8) | -0.2 (-2.6–1.8) |
| Multimorbidity | 4 | Stockholm 60–64 years Male Secondary/lower Swedish-born | 5.6 (3.6–8.0) | 7.0 (5.3–8.6) | 7.2 (5.8–8.7) | -0.2 (-1.7–0.9) |
| Multimorbidity | 5 | Stockholm 60–64 years Male Tertiary Swedish-born | 2.6 (1.2–4.5) | 4.0 (2.9–5.2) | 4.2 (3.2–5.2) | -0.1 (-1.0–0.6) |
| Multimorbidity | 6 | Göteborg 50–59 years Male Secondary/lower Swedish-born | 2.3 (1.4–3.3) | 2.8 (2.1–3.5) | 3.0 (2.4–3.6) | -0.1 (-0.8–0.4) |
| Multimorbidity | 7 | Göteborg 60–64 years Female Tertiary Swedish-born | 1.0 (0.4–1.9) | 1.7 (1.2–2.2) | 1.8 (1.4–2.2) | -0.1 (-0.5–0.2) |
| Multimorbidity | 8 | Linköping 60–64 years Female Secondary/lower Swedish-born | 2.2 (1.2–3.6) | 2.8 (2.1–3.6) | 2.9 (2.2–3.7) | -0.1 (-0.7–0.4) |
| Multimorbidity | 9 | Uppsala 60–64 years Female Tertiary Foreign-born | 2.2 (1.3–3.2) | 2.5 (1.9–3.2) | 2.6 (2.0–3.2) | -0.1 (-0.6–0.4) |
| Multimorbidity | 10 | Göteborg 60–64 years Female Secondary/lower Swedish-born | 2.5 (1.3–4.2) | 3.1 (2.3–4.0) | 3.2 (2.5–3.9) | -0.1 (-0.7–0.5) |
| Multimorbidity | 11 | Stockholm 60–64 years Male Secondary/lower Foreign-born | 5.9 (2.2–12.0) | 9.6 (7.0–12.6) | 9.7 (7.6–12.1) | -0.1 (-2.0–1.9) |
| Multimorbidity | 12 | Linköping 50–59 years Male Secondary/lower Swedish-born | 2.3 (1.5–3.4) | 2.6 (2.0–3.3) | 2.7 (2.1–3.3) | -0.1 (-0.6–0.4) |
| Multimorbidity | 13 | Uppsala 60–64 years Female Secondary/lower Foreign-born | 2.0 (0.9–3.6) | 2.7 (2.0–3.6) | 2.8 (2.2–3.5) | -0.1 (-0.7–0.5) |
| Multimorbidity | 14 | Malmö 50–59 years Female Secondary/lower Foreign-born | 1.3 (0.7–2.1) | 1.6 (1.2–2.0) | 1.6 (1.3–2.0) | -0.1 (-0.4–0.2) |
| Multimorbidity | 15 | Umeå 50–59 years Male Tertiary Swedish-born | 1.3 (0.4–2.7) | 1.9 (1.3–2.6) | 2.0 (1.5–2.6) | -0.1 (-0.5–0.3) |
| Multimorbidity | 16 | Göteborg 60–64 years Male Tertiary Foreign-born | 3.3 (0.9–7.5) | 5.7 (4.0–7.5) | 5.7 (4.4–7.3) | 0.0 (-1.2–1.1) |
| Multimorbidity | 17 | Malmö 50–59 years Female Secondary/lower Swedish-born | 1.7 (0.7–3.2) | 2.2 (1.6–2.9) | 2.2 (1.8–2.8) | 0.0 (-0.5–0.4) |
| Multimorbidity | 18 | Stockholm 60–64 years Female Secondary/lower Foreign-born | 2.6 (1.3–4.4) | 3.1 (2.3–4.0) | 3.1 (2.5–3.9) | 0.0 (-0.7–0.6) |
| Multimorbidity | 19 | Uppsala 50–59 years Male Tertiary Swedish-born | 1.2 (0.5–2.1) | 1.4 (1.0–1.9) | 1.5 (1.1–1.9) | 0.0 (-0.4–0.2) |
| Multimorbidity | 20 | Malmö 50–59 years Male Secondary/lower Swedish-born | 2.0 (1.0–3.5) | 2.3 (1.7–2.9) | 2.3 (1.8–2.9) | 0.0 (-0.5–0.4) |
| Multimorbidity | 21 | Göteborg 50–59 years Male Tertiary Swedish-born | 1.4 (0.7–2.3) | 1.6 (1.2–2.1) | 1.7 (1.3–2.1) | 0.0 (-0.4–0.3) |
| Multimorbidity | 22 | Stockholm 50–59 years Male Secondary/lower Foreign-born | 2.8 (0.9–6.3) | 4.3 (3.0–5.8) | 4.3 (3.3–5.6) | 0.0 (-0.9–0.9) |
| Multimorbidity | 23 | Göteborg 50–59 years Male Secondary/lower Foreign-born | 3.4 (1.6–5.6) | 4.0 (3.0–5.2) | 4.0 (3.2–5.1) | 0.0 (-0.9–0.7) |
| Multimorbidity | 24 | Linköping 50–59 years Male Tertiary Swedish-born | 1.4 (0.7–2.4) | 1.5 (1.1–2.0) | 1.5 (1.2–1.9) | 0.0 (-0.4–0.3) |
| Multimorbidity | 25 | Linköping 50–59 years Male Secondary/lower Foreign-born | 2.4 (0.7–5.7) | 3.6 (2.6–5.0) | 3.7 (2.8–4.8) | 0.0 (-0.8–0.8) |
| Multimorbidity | 26 | Göteborg 50–59 years Female Secondary/lower Foreign-born | 1.5 (0.6–3.1) | 1.7 (1.2–2.3) | 1.7 (1.3–2.2) | 0.0 (-0.4–0.3) |
| Multimorbidity | 27 | Stockholm 60–64 years Male Tertiary Foreign-born | 3.4 (0.9–7.6) | 5.6 (3.9–7.6) | 5.7 (4.3–7.3) | 0.0 (-1.1–1.2) |
| Multimorbidity | 28 | Linköping 60–64 years Male Tertiary Swedish-born | 3.3 (1.7–5.5) | 3.8 (2.8–4.9) | 3.8 (3.0–4.8) | 0.0 (-0.8–0.8) |
| Multimorbidity | 29 | Malmö 60–64 years Female Tertiary Swedish-born | 2.3 (0.8–4.6) | 2.9 (2.1–3.9) | 3.0 (2.3–3.7) | 0.0 (-0.7–0.6) |
| Multimorbidity | 30 | Malmö 60–64 years Male Secondary/lower Swedish-born | 8.6 (6.3–11.3) | 9.2 (7.5–11.0) | 9.2 (7.7–10.9) | 0.0 (-1.6–1.5) |
| Multimorbidity | 31 | Umeå 60–64 years Male Tertiary Foreign-born | 2.7 (0.5–8.2) | 6.6 (4.4–9.3) | 6.7 (4.7–8.9) | 0.0 (-1.3–1.3) |
| Multimorbidity | 32 | Uppsala 50–59 years Female Secondary/lower Swedish-born | 1.4 (0.4–3.4) | 1.5 (1.0–2.1) | 1.5 (1.1–2.0) | 0.0 (-0.4–0.3) |
| Multimorbidity | 33 | Stockholm 60–64 years Female Secondary/lower Swedish-born | 1.9 (0.5–4.6) | 2.4 (1.6–3.4) | 2.5 (1.8–3.2) | 0.0 (-0.5–0.5) |
| Multimorbidity | 34 | Stockholm 50–59 years Female Secondary/lower Swedish-born | 1.5 (0.5–3.4) | 1.7 (1.2–2.3) | 1.7 (1.3–2.2) | 0.0 (-0.4–0.3) |
| Multimorbidity | 35 | Uppsala 50–59 years Female Tertiary Swedish-born | 0.6 (0.2–1.2) | 0.6 (0.4–0.8) | 0.6 (0.5–0.8) | 0.0 (-0.2–0.1) |
| Multimorbidity | 36 | Stockholm 50–59 years Female Tertiary Swedish-born | 0.6 (0.2–1.3) | 0.7 (0.5–0.9) | 0.7 (0.5–0.9) | 0.0 (-0.2–0.1) |
| Multimorbidity | 37 | Umeå 60–64 years Female Secondary/lower Foreign-born | 3.4 (1.6–6.0) | 3.7 (2.7–4.9) | 3.7 (2.8–4.8) | 0.0 (-0.8–0.8) |
| Multimorbidity | 38 | Göteborg 50–59 years Female Tertiary Foreign-born | 1.1 (0.3–2.5) | 1.0 (0.7–1.3) | 1.0 (0.7–1.3) | 0.0 (-0.2–0.2) |
| Multimorbidity | 39 | Linköping 60–64 years Female Tertiary Foreign-born | 2.3 (0.5–6.3) | 2.2 (1.5–3.2) | 2.2 (1.6–3.0) | 0.0 (-0.5–0.5) |
| Multimorbidity | 40 | Malmö 50–59 years Female Tertiary Foreign-born | 1.5 (0.5–3.0) | 1.3 (0.9–1.7) | 1.3 (1.0–1.6) | 0.0 (-0.3–0.3) |
| Multimorbidity | 41 | Linköping 50–59 years Female Tertiary Foreign-born | 1.7 (0.4–4.4) | 0.9 (0.6–1.3) | 0.9 (0.6–1.2) | 0.0 (-0.2–0.2) |
| Multimorbidity | 42 | Umeå 50–59 years Female Secondary/lower Swedish-born | 2.4 (0.5–6.8) | 2.0 (1.4–2.9) | 2.0 (1.5–2.7) | 0.0 (-0.4–0.5) |
| Multimorbidity | 43 | Umeå 60–64 years Female Tertiary Swedish-born | 2.3 (0.4–6.2) | 2.7 (1.8–3.8) | 2.7 (1.9–3.7) | 0.0 (-0.6–0.6) |
| Multimorbidity | 44 | Stockholm 60–64 years Female Tertiary Swedish-born | 2.4 (0.9–4.8) | 2.3 (1.6–3.1) | 2.3 (1.7–3.0) | 0.0 (-0.4–0.5) |
| Multimorbidity | 45 | Umeå 50–59 years Female Tertiary Foreign-born | 2.0 (0.4–5.1) | 1.2 (0.7–1.7) | 1.2 (0.8–1.6) | 0.0 (-0.2–0.3) |
| Multimorbidity | 46 | Linköping 50–59 years Female Secondary/lower Foreign-born | 2.3 (0.6–5.6) | 1.6 (1.1–2.2) | 1.6 (1.2–2.1) | 0.0 (-0.3–0.4) |
| Multimorbidity | 47 | Uppsala 50–59 years Female Tertiary Foreign-born | 1.8 (0.5–3.9) | 0.9 (0.6–1.2) | 0.9 (0.6–1.1) | 0.0 (-0.2–0.2) |
| Multimorbidity | 48 | Uppsala 60–64 years Female Secondary/lower Swedish-born | 2.2 (0.6–5.2) | 2.2 (1.5–3.0) | 2.2 (1.6–2.9) | 0.0 (-0.4–0.5) |
| Multimorbidity | 49 | Stockholm 50–59 years Female Tertiary Foreign-born | 1.8 (0.6–3.8) | 1.0 (0.7–1.4) | 1.0 (0.7–1.3) | 0.0 (-0.2–0.3) |
| Multimorbidity | 50 | Umeå 50–59 years Female Tertiary Swedish-born | 1.4 (0.6–2.6) | 0.8 (0.6–1.2) | 0.8 (0.6–1.1) | 0.0 (-0.2–0.2) |
| Multimorbidity | 51 | Linköping 50–59 years Female Tertiary Swedish-born | 1.1 (0.5–1.9) | 0.7 (0.5–0.9) | 0.6 (0.5–0.8) | 0.0 (-0.1–0.2) |
| Multimorbidity | 52 | Göteborg 50–59 years Female Tertiary Swedish-born | 1.1 (0.6–1.8) | 0.7 (0.5–1.0) | 0.7 (0.5–0.9) | 0.0 (-0.1–0.2) |
| Multimorbidity | 53 | Malmö 50–59 years Male Tertiary Swedish-born | 2.2 (1.2–3.5) | 2.2 (1.6–2.8) | 2.2 (1.7–2.7) | 0.0 (-0.4–0.5) |
| Multimorbidity | 54 | Umeå 50–59 years Male Secondary/lower Swedish-born | 2.3 (1.0–4.5) | 2.1 (1.5–2.9) | 2.1 (1.6–2.7) | 0.0 (-0.4–0.5) |
| Multimorbidity | 55 | Umeå 50–59 years Male Tertiary Foreign-born | 3.1 (0.8–7.8) | 4.7 (3.3–6.6) | 4.7 (3.5–6.2) | 0.0 (-0.9–1.1) |
| Multimorbidity | 56 | Linköping 60–64 years Female Secondary/lower Foreign-born | 3.7 (0.8–10.0) | 4.0 (2.7–5.5) | 3.9 (2.9–5.2) | 0.0 (-0.8–0.9) |
| Multimorbidity | 57 | Malmö 60–64 years Female Secondary/lower Foreign-born | 4.1 (2.5–6.1) | 4.1 (3.2–5.2) | 4.1 (3.3–4.9) | 0.0 (-0.7–0.8) |
| Multimorbidity | 58 | Linköping 50–59 years Male Tertiary Foreign-born | 3.0 (0.9–6.7) | 2.1 (1.5–3.0) | 2.1 (1.5–2.8) | 0.0 (-0.4–0.5) |
| Multimorbidity | 59 | Linköping 50–59 years Female Secondary/lower Swedish-born | 1.6 (0.9–2.5) | 1.2 (0.9–1.6) | 1.1 (0.9–1.5) | 0.0 (-0.2–0.3) |
| Multimorbidity | 60 | Uppsala 60–64 years Female Tertiary Swedish-born | 2.8 (1.0–5.9) | 2.1 (1.4–2.8) | 2.0 (1.5–2.6) | 0.0 (-0.4–0.6) |
| Multimorbidity | 61 | Malmö 60–64 years Female Secondary/lower Swedish-born | 3.5 (1.3–6.9) | 3.2 (2.3–4.3) | 3.2 (2.4–4.0) | 0.0 (-0.6–0.7) |
| Multimorbidity | 62 | Uppsala 50–59 years Male Secondary/lower Swedish-born | 2.1 (1.1–3.6) | 1.6 (1.2–2.1) | 1.6 (1.2–2.0) | 0.0 (-0.3–0.4) |
| Multimorbidity | 63 | Malmö 50–59 years Female Tertiary Swedish-born | 1.5 (0.8–2.5) | 0.9 (0.7–1.3) | 0.9 (0.7–1.1) | 0.0 (-0.1–0.3) |
| Multimorbidity | 64 | Göteborg 60–64 years Female Tertiary Foreign-born | 3.5 (1.1–7.6) | 2.5 (1.8–3.4) | 2.5 (1.9–3.2) | 0.0 (-0.5–0.6) |
| Multimorbidity | 65 | Linköping 60–64 years Female Tertiary Swedish-born | 2.2 (1.0–3.8) | 1.7 (1.2–2.2) | 1.6 (1.3–2.1) | 0.0 (-0.3–0.5) |
| Multimorbidity | 66 | Malmö 60–64 years Female Tertiary Foreign-born | 3.8 (2.7–5.2) | 3.8 (3.1–4.7) | 3.8 (3.1–4.5) | 0.0 (-0.6–0.7) |
| Multimorbidity | 67 | Uppsala 50–59 years Female Secondary/lower Foreign-born | 1.6 (0.8–2.8) | 1.1 (0.8–1.6) | 1.1 (0.8–1.4) | 0.0 (-0.2–0.3) |
| Multimorbidity | 68 | Malmö 50–59 years Male Secondary/lower Foreign-born | 5.1 (2.5–8.7) | 5.6 (4.2–7.3) | 5.5 (4.4–6.9) | 0.0 (-1.0–1.3) |
| Multimorbidity | 69 | Malmö 50–59 years Male Tertiary Foreign-born | 5.0 (3.0–7.5) | 5.2 (4.0–6.6) | 5.2 (4.2–6.3) | 0.0 (-0.9–1.1) |
| Multimorbidity | 70 | Göteborg 50–59 years Male Tertiary Foreign-born | 3.3 (1.4–6.3) | 2.3 (1.7–3.2) | 2.3 (1.7–3.0) | 0.0 (-0.4–0.7) |
| Multimorbidity | 71 | Stockholm 50–59 years Male Secondary/lower Swedish-born | 2.4 (1.2–4.2) | 1.8 (1.3–2.5) | 1.8 (1.4–2.3) | 0.0 (-0.3–0.5) |
| Multimorbidity | 72 | Umeå 60–64 years Male Tertiary Swedish-born | 4.7 (1.9–8.9) | 4.9 (3.5–6.7) | 4.9 (3.7–6.3) | 0.0 (-0.9–1.2) |
| Multimorbidity | 73 | Stockholm 50–59 years Female Secondary/lower Foreign-born | 1.9 (1.1–3.0) | 1.3 (1.0–1.8) | 1.3 (1.0–1.6) | 0.0 (-0.2–0.4) |
| Multimorbidity | 74 | Umeå 50–59 years Female Secondary/lower Foreign-born | 2.4 (1.2–4.0) | 1.5 (1.1–2.1) | 1.5 (1.1–1.9) | 0.1 (-0.2–0.5) |
| Multimorbidity | 75 | Göteborg 60–64 years Male Tertiary Swedish-born | 4.3 (2.6–6.5) | 4.2 (3.2–5.4) | 4.2 (3.3–5.1) | 0.1 (-0.7–1.0) |
| Multimorbidity | 76 | Linköping 60–64 years Male Tertiary Foreign-born | 4.3 (1.2–10.8) | 5.2 (3.6–7.2) | 5.2 (3.8–6.8) | 0.1 (-1.0–1.3) |
| Multimorbidity | 77 | Uppsala 50–59 years Male Secondary/lower Foreign-born | 4.3 (1.4–9.3) | 3.9 (2.7–5.4) | 3.8 (2.8–5.0) | 0.1 (-0.7–1.0) |
| Multimorbidity | 78 | Umeå 60–64 years Female Secondary/lower Swedish-born | 5.1 (1.3–12.8) | 3.0 (1.9–4.3) | 2.9 (2.0–4.0) | 0.1 (-0.5–0.8) |
| Multimorbidity | 79 | Göteborg 50–59 years Female Secondary/lower Swedish-born | 1.9 (1.2–2.9) | 1.3 (1.0–1.8) | 1.3 (1.0–1.6) | 0.1 (-0.2–0.4) |
| Multimorbidity | 80 | Umeå 50–59 years Male Secondary/lower Foreign-born | 4.6 (1.0–13.4) | 5.1 (3.5–7.4) | 5.1 (3.6–6.8) | 0.1 (-1.0–1.4) |
| Multimorbidity | 81 | Göteborg 60–64 years Male Secondary/lower Swedish-born | 7.2 (5.2–9.7) | 7.3 (5.9–8.9) | 7.2 (5.9–8.7) | 0.1 (-1.2–1.4) |
| Multimorbidity | 82 | Stockholm 50–59 years Male Tertiary Foreign-born | 4.6 (2.1–8.0) | 4.1 (3.0–5.5) | 4.0 (3.1–5.1) | 0.1 (-0.6–1.0) |
| Multimorbidity | 83 | Uppsala 60–64 years Male Tertiary Swedish-born | 4.2 (2.4–6.6) | 3.8 (2.8–4.9) | 3.7 (2.9–4.6) | 0.1 (-0.5–1.0) |
| Multimorbidity | 84 | Malmö 60–64 years Male Tertiary Foreign-born | 6.5 (2.8–12.0) | 7.4 (5.5–9.6) | 7.3 (5.7–9.1) | 0.1 (-1.2–1.8) |
| Multimorbidity | 85 | Uppsala 50–59 years Male Tertiary Foreign-born | 5.1 (2.1–9.5) | 3.7 (2.6–5.0) | 3.6 (2.7–4.6) | 0.1 (-0.5–1.1) |
| Multimorbidity | 86 | Umeå 60–64 years Female Tertiary Foreign-born | 4.0 (2.6–5.9) | 3.6 (2.7–4.7) | 3.5 (2.7–4.4) | 0.1 (-0.5–1.0) |
| Multimorbidity | 87 | Uppsala 60–64 years Male Tertiary Foreign-born | 6.2 (2.2–12.5) | 5.2 (3.7–7.0) | 5.0 (3.7–6.5) | 0.1 (-0.8–1.5) |
| Multimorbidity | 88 | Linköping 60–64 years Male Secondary/lower Swedish-born | 6.9 (4.9–9.2) | 6.7 (5.3–8.4) | 6.6 (5.3–8.1) | 0.1 (-1.0–1.4) |
| Multimorbidity | 89 | Stockholm 50–59 years Male Tertiary Swedish-born | 2.9 (1.7–4.3) | 1.8 (1.3–2.4) | 1.7 (1.3–2.1) | 0.1 (-0.2–0.7) |
| Multimorbidity | 90 | Stockholm 60–64 years Female Tertiary Foreign-born | 3.5 (2.4–4.9) | 3.1 (2.4–3.9) | 2.9 (2.4–3.6) | 0.1 (-0.3–0.9) |
| Multimorbidity | 91 | Linköping 60–64 years Male Secondary/lower Foreign-born | 7.0 (2.0–16.3) | 9.0 (6.5–12.4) | 8.9 (6.8–11.3) | 0.1 (-1.6–2.4) |
| Multimorbidity | 92 | Umeå 60–64 years Male Secondary/lower Foreign-born | 7.5 (1.6–21.3) | 11.5 (8.1–15.8) | 11.3 (8.4–14.6) | 0.2 (-1.8–3.0) |
| Multimorbidity | 93 | Göteborg 60–64 years Female Secondary/lower Foreign-born | 6.9 (3.3–11.7) | 4.6 (3.3–6.3) | 4.3 (3.3–5.5) | 0.2 (-0.5–1.6) |
| Multimorbidity | 94 | Uppsala 60–64 years Male Secondary/lower Swedish-born | 7.3 (5.0–10.0) | 6.6 (5.2–8.4) | 6.4 (5.1–7.9) | 0.2 (-0.8–1.8) |
| Multimorbidity | 95 | Malmö 60–64 years Male Tertiary Swedish-born | 6.8 (4.3–10.0) | 5.6 (4.4–7.4) | 5.4 (4.3–6.5) | 0.3 (-0.6–1.8) |
| Multimorbidity | 96 | Göteborg 60–64 years Male Secondary/lower Foreign-born | 12.4 (7.1–19.3) | 10.2 (7.8–13.4) | 9.8 (7.7–12.1) | 0.5 (-1.0–3.0) |

Table S7. Parameters estimate from different intersectional models based on social strata

| Variables | **Any CMD** | |  | **Cardiometabolic multimorbidity** | |
| --- | --- | --- | --- | --- | --- |
|  | Model 2 | Model 3 |  | Model 2 | Model 3 |
|  | Partially adjusted model | Intersectional interaction model |  | Partially adjusted model | Intersectional interaction model |
|  | OR (95% CI) | OR (95% CI) |  | OR (95% CI) | OR (95% CI) |
| **Measures of association (fixed effects)** | | | |  |  |
| Age (ref. 50–59 years) |  |  |  |  |  |
| 60–64 years | 1.60 (1.05 to 2.42) | 1.76 (1.62 to 1.91) |  | 2.42 (1.31 to 4.46) | 2.60 (2.18 to 3.12) |
| Sex (ref. Female) |  |  |  |  |  |
| Male | 1.65 (1.19 to 2.29) | 1.49 (1.37 to 1.63) |  | 2.35 (1.10 to 5.05) | 2.40 (1.99 to 2.89) |
| Educational attainment (ref. Tertiary) | | | |  |  |
| Secondary/lower | 1.71 (1.04 to 2.83) | 1.48 (1.37 to 1.60) |  | 1.93 (0.98 to 3.84) | 1.83 (1.52 to 2.20) |
| Country of birth (ref. Swedish-born) | | | |  |  |
| Foreign-born | 1.29 (0.77 to 2.16) | 1.34 (1.22 to 1.46) |  | 1.18 (0.49 to 2.87) | 1.43 (1.16 to 1.77) |
| **Measures of variance (random effects)** | | | |  |  |
| Stratum-level | 0.14 (0.06 to 0.30)(a) 0.18 (0.08 to 0.37)(b) 0.20 (0.08 to 0.45)(c) 0.21 (0.10 to 0.46)(d) | 0.00 (0.00 to 0.01) |  | 0.45 (0.19 to 0.98)(a) 0.52 (0.22 to 1.13)(b) 0.65 (0.28 to 1.41)(c) 0.77 (0.32 to 1.69)(d) | 0.01 (0.00 to 0.05) |
| VPC | 4.01 (1.82 to 8.37)(a) 5.04 (2.39 to 10.17)(b) 5.70 (2.48 to 12.03)(c) 6.03 (2.81 to 12.24)(d) | 0.10 (0.01 to 0.38) |  | 11.74 (5.44 to 22.97)(a) 13.30 (6.25 to 25.60)(b) 16.12 (7.83 to 29.99)(c) 18.40 (8.98 to 33.94)(d) | 0.28 (0.02 to 1.42) |
| PCV | 39.49%(a)  23.12%(b)  12.04%(c)  6.72%(d) | 98.57% |  | 40.18%(a)  30.81%(b)  12.89%(c)  -2.73%(d) | 98.74% |
| **Discriminatory accuracy** | | | | | |
| AUC | 0.61 (0.60–0.62) | 0.61 (0.60–0.62) |  | 0.69 (0.68–0.71) | 0.69 (0.68–0.71) |

**Notes:**

CMD: cardiometabolic disease, OR: odds ratio, CI: confidence interval

Model 2: Individuals (level 1) nested within 16 sociodemographic strata (level 2), with one variable (a: age, b: sex, c: education, d: country of birth) included at a time in the fixed effects.

Model 3: Individuals (level 1) nested within 16 sociodemographic strata (level 2), with all variables included in the fixed effects.

VPC = variance partition coefficient, indicates the percent of the total variation in the dependent variable that is attributable to the between–stratum level; PVC: proportional change in the variance, indicates the percent of the total between-stratum variance from the null model (Model 1B) that was explained after adjustment for additive main effects, AUC = area under the receiver operating characteristics curve, indicates the accuracy of sociodemographic strata in discriminating individuals with cardiometabolic multimorbidity from those without cardiometabolic multimorbidity.

Table S8. The predicted prevalences of any CMD and cardiometabolic multimorbidity based on main effect and interaction effects, sorted by social strata (ranks are sorted in ascending order by Model 3, interaction effect)

| **Outcome** | **Rank** | **Social strata**  **(Age, sex, education, country of birth)** | **Model 1** | **Model 3 (main effect+interaction)** | **Model 3 (main effect)** | **Model 3 (interaction)** |
| --- | --- | --- | --- | --- | --- | --- |
| Any CMD | 1 | 60–64 years Female Tertiary Foreign-born | 27.8 (23.6 to 32.4) | 30.2 (27.3 to 32.9) | 30.7 (28.4 to 33.3) | -0.6 (-3.3 to 1.5) |
| Any CMD | 2 | 60–64 years Female Secondary/lower Swedish-born | 31.9 (30.0 to 34.0) | 32.4 (30.7 to 34.1) | 33.0 (31.0 to 35.0) | -0.5 (-2.7 to 1.3) |
| Any CMD | 3 | 50–59 years Male Tertiary Swedish-born | 21.1 (19.8 to 22.5) | 21.5 (20.2 to 22.8) | 22.0 (20.3 to 23.5) | -0.5 (-2.1 to 1.0) |
| Any CMD | 4 | 50–59 years Female Secondary/lower Foreign-born | 25.8 (23.0 to 28.7) | 26.7 (24.5 to 28.8) | 27.2 (25.2 to 29.3) | -0.4 (-2.6 to 1.3) |
| Any CMD | 5 | 50–59 years Male Secondary/lower Foreign-born | 34.5 (31.4 to 37.6) | 35.4 (33.0 to 37.7) | 35.7 (33.4 to 38.0) | -0.4 (-2.7 to 1.7) |
| Any CMD | 6 | 60–64 years Female Tertiary Swedish-born | 24.2 (22.4 to 26.0) | 24.6 (23.0 to 26.1) | 24.9 (23.3 to 26.6) | -0.4 (-2.2 to 1.1) |
| Any CMD | 7 | 60–64 years Male Tertiary Foreign-born | 38.7 (33.2 to 44.2) | 39.7 (36.5 to 43.0) | 39.8 (37.2 to 42.4) | -0.1 (-2.6 to 2.4) |
| Any CMD | 8 | 50–59 years Male Secondary/lower Swedish-born | 29.3 (28.0 to 30.6) | 29.3 (28.1 to 30.6) | 29.4 (27.6 to 31.1) | -0.1 (-1.8 to 1.7) |
| Any CMD | 9 | 50–59 years Female Secondary/lower Swedish-born | 22.3 (21.1 to 23.5) | 22.1 (21.0 to 23.2) | 21.8 (20.4 to 23.2) | 0.2 (-1.2 to 1.8) |
| Any CMD | 10 | 50–59 years Male Tertiary Foreign-born | 28.4 (24.9 to 31.8) | 27.6 (25.3 to 30.0) | 27.3 (25.2 to 29.4) | 0.3 (-1.6 to 2.4) |
| Any CMD | 11 | 60–64 years Male Tertiary Swedish-born | 33.7 (31.5 to 36.1) | 33.4 (31.6 to 35.3) | 33.1 (31.2 to 35.0) | 0.3 (-1.6 to 2.5) |
| Any CMD | 12 | 50–59 years Female Tertiary Foreign-born | 21.6 (18.8 to 24.5) | 20.4 (18.7 to 22.4) | 20.2 (18.5 to 21.9) | 0.3 (-1.2 to 2.2) |
| Any CMD | 13 | 60–64 years Female Secondary/lower Foreign-born | 40.9 (36.3 to 45.7) | 40.0 (37.2 to 43.1) | 39.6 (37.0 to 42.5) | 0.4 (-1.9 to 3.0) |
| Any CMD | 14 | 60–64 years Male Secondary/lower Swedish-born | 42.9 (40.9 to 44.9) | 42.7 (40.9 to 44.5) | 42.3 (40.2 to 44.6) | 0.4 (-1.7 to 2.6) |
| Any CMD | 15 | 60–64 years Male Secondary/lower Foreign-born | 50.8 (45.8 to 55.9) | 50.0 (46.9 to 53.5) | 49.4 (46.9 to 52.1) | 0.5 (-1.8 to 3.6) |
| Any CMD | 16 | 50–59 years Female Tertiary Swedish-born | 17.1 (16.0 to 18.3) | 16.5 (15.5 to 17.6) | 15.9 (14.6 to 17.0) | 0.7 (-0.4 to 2.2) |
| Multimorbidity | 1 | 60–64 years Female Secondary/lower Swedish-born | 2.8 (2.2 to 3.6) | 3.2 (2.6 to 3.8) | 3.3 (2.8 to 4.0) | -0.2 (-0.8 to 0.3) |
| Multimorbidity | 2 | 60–64 years Male Secondary/lower Swedish-born | 7.4 (6.4 to 8.4) | 7.5 (6.6 to 8.4) | 7.6 (6.5 to 9.1) | -0.1 (-1.6 to 0.8) |
| Multimorbidity | 3 | 50–59 years Female Secondary/lower Foreign-born | 1.2 (0.7 to 2.0) | 1.8 (1.3 to 2.3) | 1.9 (1.4 to 2.4) | -0.1 (-0.4 to 0.2) |
| Multimorbidity | 4 | 50–59 years Male Secondary/lower Swedish-born | 3.0 (2.5 to 3.5) | 3.0 (2.6 to 3.4) | 3.1 (2.6 to 3.6) | -0.1 (-0.6 to 0.3) |
| Multimorbidity | 5 | 50–59 years Male Tertiary Swedish-born | 1.6 (1.2 to 2.1) | 1.7 (1.4 to 2.0) | 1.7 (1.4 to 2.1) | 0.0 (-0.3 to 0.2) |
| Multimorbidity | 6 | 60–64 years Male Secondary/lower Foreign-born | 10.0 (7.3 to 13.2) | 10.5 (8.6 to 12.8) | 10.6 (8.7 to 12.8) | 0.0 (-1.7 to 1.5) |
| Multimorbidity | 7 | 50–59 years Female Tertiary Foreign-born | 0.9 (0.4 to 1.7) | 1.0 (0.7 to 1.3) | 1.0 (0.8 to 1.3) | 0.0 (-0.2 to 0.2) |
| Multimorbidity | 8 | 60–64 years Female Tertiary Swedish-born | 1.8 (1.3 to 2.4) | 1.8 (1.5 to 2.2) | 1.8 (1.5 to 2.3) | 0.0 (-0.3 to 0.3) |
| Multimorbidity | 9 | 60–64 years Male Tertiary Foreign-born | 5.8 (3.5 to 8.7) | 6.1 (4.7 to 7.7) | 6.1 (4.8 to 7.6) | 0.0 (-1.0 to 1.1) |
| Multimorbidity | 10 | 50–59 years Female Tertiary Swedish-born | 0.8 (0.6 to 1.1) | 0.7 (0.6 to 0.9) | 0.7 (0.6 to 0.9) | 0.0 (-0.1 to 0.2) |
| Multimorbidity | 11 | 60–64 years Female Tertiary Foreign-born | 2.9 (1.6 to 4.8) | 2.7 (1.9 to 3.5) | 2.6 (2.0 to 3.3) | 0.0 (-0.4 to 0.6) |
| Multimorbidity | 12 | 50–59 years Male Tertiary Foreign-born | 2.6 (1.6 to 3.9) | 2.5 (1.9 to 3.2) | 2.4 (1.9 to 3.0) | 0.0 (-0.4 to 0.5) |
| Multimorbidity | 13 | 60–64 years Male Tertiary Swedish-born | 4.5 (3.6 to 5.6) | 4.4 (3.7 to 5.2) | 4.3 (3.6 to 5.3) | 0.0 (-0.7 to 0.8) |
| Multimorbidity | 14 | 50–59 years Male Secondary/lower Foreign-born | 4.6 (3.3 to 6.0) | 4.4 (3.5 to 5.4) | 4.3 (3.5 to 5.2) | 0.1 (-0.6 to 0.8) |
| Multimorbidity | 15 | 50–59 years Female Secondary/lower Swedish-born | 1.7 (1.3 to 2.1) | 1.4 (1.1 to 1.7) | 1.3 (1.1 to 1.6) | 0.1 (-0.1 to 0.4) |
| Multimorbidity | 16 | 60–64 years Female Secondary/lower Foreign-born | 5.8 (3.8 to 8.1) | 4.9 (3.8 to 6.3) | 4.7 (3.7 to 5.9) | 0.2 (-0.5 to 1.3) |

Table S9. Agreement of number of cardiometabolic disease: self-report vs combined sources (self-report, registers, biomarkers) by intersectional strata

| **Intersectional strata** | **Agreement (%)** | **Disagreement (%)** |
| --- | --- | --- |
| 50–59 years Female Tertiary Swedish-born | 85.3 | 14.7 |
| 50–59 years Male Tertiary Swedish-born | 83.0 | 17.0 |
| 50–59 years Female Tertiary Foreign-born | 82.6 | 17.4 |
| 50–59 years Female Secondary/lower Swedish-born | 82.6 | 17.4 |
| 50–59 years Female Secondary/lower Foreign-born | 80.0 | 20.0 |
| 60–64 years Female Tertiary Swedish-born | 79.9 | 20.1 |
| 50–59 years Male Tertiary Foreign-born | 79.4 | 20.6 |
| 50–59 years Male Secondary/lower Swedish-born | 77.7 | 22.3 |
| 60–64 years Female Tertiary Foreign-born | 77.6 | 22.4 |
| 60–64 years Male Tertiary Swedish-born | 75.2 | 24.8 |
| 60–64 years Female Secondary/lower Swedish-born | 75.1 | 24.9 |
| 60–64 years Male Tertiary Foreign-born | 74.7 | 25.3 |
| 50–59 years Male Secondary/lower Foreign-born | 74.0 | 26.0 |
| 60–64 years Male Secondary/lower Swedish-born | 70.3 | 29.7 |
| 60–64 years Female Secondary/lower Foreign-born | 69.0 | 31.0 |
| 60–64 years Male Secondary/lower Foreign-born | 64.5 | 35.5 |

**Notes:**

Agreement = exact match in the number of cardiometabolic conditions between self-report and the combined data source (self-report + registers + biomarkers). Disagreement= any mismatch. Values shown are row percentages.

Table S10. Sensitivity analysis: predicted prevalence of type 2 diabetes and CVD (heart disease and stroke) by social strata, based on Model 1 (strata are sorted in ascending order by the prevalence of heart disease and stroke)

| **Social strata (Age, Sex, Education, Country of birth)** | **n** | **Type 2 diabetes** | **Heart disease and stroke** |
| --- | --- | --- | --- |
|  |  | Predicted prevalence (%, 95% CI) | |
| 50–59 years Female Tertiary Swedish-born | 4139 | 15.0 (14.0–16.1) | 2.9 (2.4–3.4) |
| 50–59 years Male Tertiary Swedish-born | 3209 | 18.6 (17.1–20.0) | 4.1 (3.4–4.8) |
| 50–59 years Female Tertiary Foreign-born | 759 | 18.3 (15.6–21.2) | 4.3 (3.0–5.8) |
| 50–59 years Female Secondary/lower Swedish-born | 4165 | 19.5 (18.3–20.7) | 4.4 (3.8–5.0) |
| 50–59 years Female Secondary/lower Foreign-born | 927 | 22.3 (19.8–25.0) | 4.6 (3.4–6.0) |
| 60–64 years Female Tertiary Swedish-born | 2133 | 20.8 (19.1–22.5) | 5.2 (4.3–6.1) |
| 50–59 years Male Secondary/lower Swedish-born | 4605 | 25.4 (24.1–26.7) | 6.6 (5.9–7.4) |
| 50–59 years Male Tertiary Foreign-born | 631 | 24.2 (21.0–27.4) | 6.8 (5.1–8.9) |
| 60–64 years Female Secondary/lower Swedish-born | 2101 | 27.1 (25.2–29.0) | 7.6 (6.5–8.7) |
| 60–64 years Female Tertiary Foreign-born | 379 | 22.2 (18.3–26.5) | 8.0 (5.6–10.9) |
| 50–59 years Male Secondary/lower Foreign-born | 907 | 30.6 (27.7–33.6) | 8.2 (6.5–10.1) |
| 60–64 years Male Tertiary Swedish-born | 1594 | 28.3 (26.2–30.6) | 9.7 (8.3–11.3) |
| 60–64 years Male Tertiary Foreign-born | 289 | 33.3 (28.0–38.9) | 10.9 (7.8–14.6) |
| 60–64 years Female Secondary/lower Foreign-born | 429 | 33.5 (29.2–38.0) | 13.0 (10.0–16.1) |
| 60–64 years Male Secondary/lower Swedish-born | 2457 | 36.3 (34.4–38.1) | 13.7 (12.4–15.1) |
| 60–64 years Male Secondary/lower Foreign-born | 369 | 44.1 (39.2–49.1) | 16.2 (12.7–20.1) |

Table S11. Sensitivity analysis: predicted prevalence of any CMD and cardiometabolic multimorbidity by social strata^1^, based on Model 1 (N=29,065)

| **Intersectional social strata (Age, Sex, Employment, Country of birth)** | **n** | **Any CMD** | **Cardiometabolic multimorbidity** |
| --- | --- | --- | --- |
|  |  | Predicted prevalence (%, 95% CI) | |
| 50–59 years Female Employed Foreign-born | 1324 | 21.9 (19.7–24.2) | 0.7 (0.4–1.3) |
| 50–59 years Female Employed Swedish-born | 7662 | 18.6 (17.8–19.5) | 0.9 (0.7–1.2) |
| 60–64 years Female Employed Swedish-born | 3412 | 26.9 (25.4–28.3) | 1.8 (1.4–2.3) |
| 50–59 years Male Employed Swedish-born | 7374 | 25.0 (24.1–26.0) | 2.0 (1.7–2.4) |
| 50–59 years Female Unemployed Foreign-born | 354 | 31.1 (26.7–35.9) | 2.2 (1.0–3.8) |
| 60–64 years Female Employed Foreign-born | 542 | 30.0 (26.3–33.7) | 2.7 (1.6–4.1) |
| 50–59 years Male Employed Foreign-born | 1250 | 29.5 (27.0–32.1) | 2.8 (2.0–3.8) |
| 60–64 years Female Unemployed Swedish-born | 824 | 32.8 (29.6–35.9) | 4.3 (3.0–5.7) |
| 50–59 years Female Unemployed Swedish-born | 636 | 31.5 (28.1–35.1) | 4.5 (3.1–6.3) |
| 60–64 years Male Employed Swedish-born | 3283 | 37.2 (35.6–38.9) | 4.9 (4.2–5.7) |
| 60–64 years Male Employed Foreign-born | 469 | 43.9 (39.3–48.5) | 5.1 (3.3–7.2) |
| 50–59 years Male Unemployed Foreign-born | 281 | 42.8 (37.3–48.6) | 7.5 (4.7–10.7) |
| 60–64 years Female Unemployed Foreign-born | 261 | 45.3 (39.8–50.9) | 8.4 (5.5–12.0) |
| 50–59 years Male Unemployed Swedish-born | 438 | 41.5 (37.2–45.8) | 8.7 (6.3–11.5) |
| 60–64 years Male Unemployed Swedish-born | 764 | 47.7 (44.1–51.3) | 11.7 (9.5–14.1) |
| 60–64 years Male Unemployed Foreign-born | 191 | 50.1 (43.2–57.0) | 15.8 (11.1–21.2) |

**Notes:**

^1^Intersectional social strata was constructed based on the combination of age groups, sex, employment status, and country of birth. Employment status was defined as employed or unemployed at the time of the survey.

The analytic sample for this sensitivity analysis (N = 29,065) differs slightly from the main analysis (N = 29,093) due to missing data on employment status.

Table S12. Sensitivity analysis: predicted prevalence of any CMD and cardiometabolic multimorbidity by social strata^1^, based on Model 1 (N=28,351)

| **Social strata (Age, Sex, Education, Country of birth**^1^**)** | **n** | **Any CMD** | **Cardiometabolic multimorbidity** |
| --- | --- | --- | --- |
|  |  | Predicted prevalence (%, 95% CI) | |
| 50–59 years Female Tertiary Swedish-born | 3981 | 17.2 (16.0–18.4) | 0.8 (0.6–1.1) |
| 50–59 years Female Tertiary Foreign-born | 703 | 21.6 (18.7–24.7) | 1.0 (0.4–1.8) |
| 50–59 years Female Secondary/lower Foreign-born | 900 | 25.8 (23.1–28.7) | 1.3 (0.7–2.0) |
| 50–59 years Male Tertiary Swedish-born | 3087 | 21.2 (19.7–22.6) | 1.6 (1.2–2.0) |
| 50–59 years Female Secondary/lower Swedish-born | 3901 | 22.1 (20.9–23.4) | 1.6 (1.2–2.0) |
| 60–64 years Female Tertiary Swedish-born | 2056 | 24.0 (22.2–25.8) | 1.7 (1.2–2.3) |
| 50–59 years Female Tertiary Second-generation migrant | 121 | 21.2 (15.0–28.1) | 2.2 (0.6–4.7) |
| 50–59 years Male Tertiary Foreign-born | 586 | 28.2 (24.6–31.7) | 2.6 (1.5–3.9) |
| 50–59 years Female Secondary/lower Second-generation migrant | 154 | 26.5 (20.5–33.1) | 2.7 (1.1–5.4) |
| 60–64 years Female Secondary/lower Swedish-born | 2000 | 31.5 (29.4–33.6) | 2.8 (2.1–3.6) |
| 60–64 years Female Tertiary Foreign-born | 356 | 27.3 (23.2–31.9) | 2.8 (1.5–4.6) |
| 50–59 years Male Tertiary Second-generation migrant | 108 | 22.4 (16.2–29.4) | 2.9 (1.0–5.8) |
| 50–59 years Male Secondary/lower Swedish-born | 4316 | 29.0 (27.7–30.4) | 3.0 (2.5–3.5) |
| 50–59 years Male Secondary/lower Second-generation migrant | 153 | 29.9 (23.5–36.9) | 3.2 (1.4–6.0) |
| 60–64 years Male Tertiary Second-generation migrant | 43 | 29.8 (19.8–41.0) | 3.8 (1.1–9.1) |
| 60–64 years Female Tertiary Second-generation migrant | 42 | 28.8 (19.0–40.1) | 4.0 (1.1–9.2) |
| 60–64 years Female Secondary/lower Second-generation migrant | 40 | 34.2 (23.3–46.5) | 4.0 (1.1–9.5) |
| 50–59 years Male Secondary/lower Foreign-born | 872 | 34.1 (31.0–37.2) | 4.5 (3.3–6.0) |
| 60–64 years Male Tertiary Swedish-born | 1525 | 33.5 (31.2–35.9) | 4.6 (3.7–5.7) |
| 60–64 years Female Secondary/lower Foreign-born | 411 | 40.8 (36.2–45.6) | 5.8 (3.8–8.2) |
| 60–64 years Male Tertiary Foreign-born | 274 | 38.8 (33.4–44.6) | 5.8 (3.4–8.7) |
| 60–64 years Male Secondary/lower Swedish-born | 2322 | 42.6 (40.7–44.5) | 7.1 (6.0–8.1) |
| 60–64 years Male Secondary/lower Second-generation migrant | 49 | 39.4 (28.7–51.1) | 9.9 (4.0–18.7) |
| 60–64 years Male Secondary/lower Foreign-born | 351 | 50.5 (45.5–55.8) | 9.9 (7.0–13.2) |

**Notes:**

^1^Intersectional social strata were constructed from age group, sex, educational attainment, and country of birth (three categories). Country of birth was classified using parental country of birth into: (i) Swedish-born—participants born in or outside Sweden with at least one Swedish-born parent (n = 23,188); (ii) second-generation migrant—participants born in Sweden with two foreign-born parents (n = 710); and (iii) foreign-born—participants born outside Sweden with two foreign-born parents (n = 4,453). The analytic sample for this sensitivity analysis (N = 28,351) differs slightly from the main analysis (N = 29,093) due to missing data on parents’ country of birth.

Figure S1. Sample flowchart

Figure S2. Intersectional interaction effects on prevalence of any CMD and cardiometabolic multimorbidity based on Model 3, by social strata


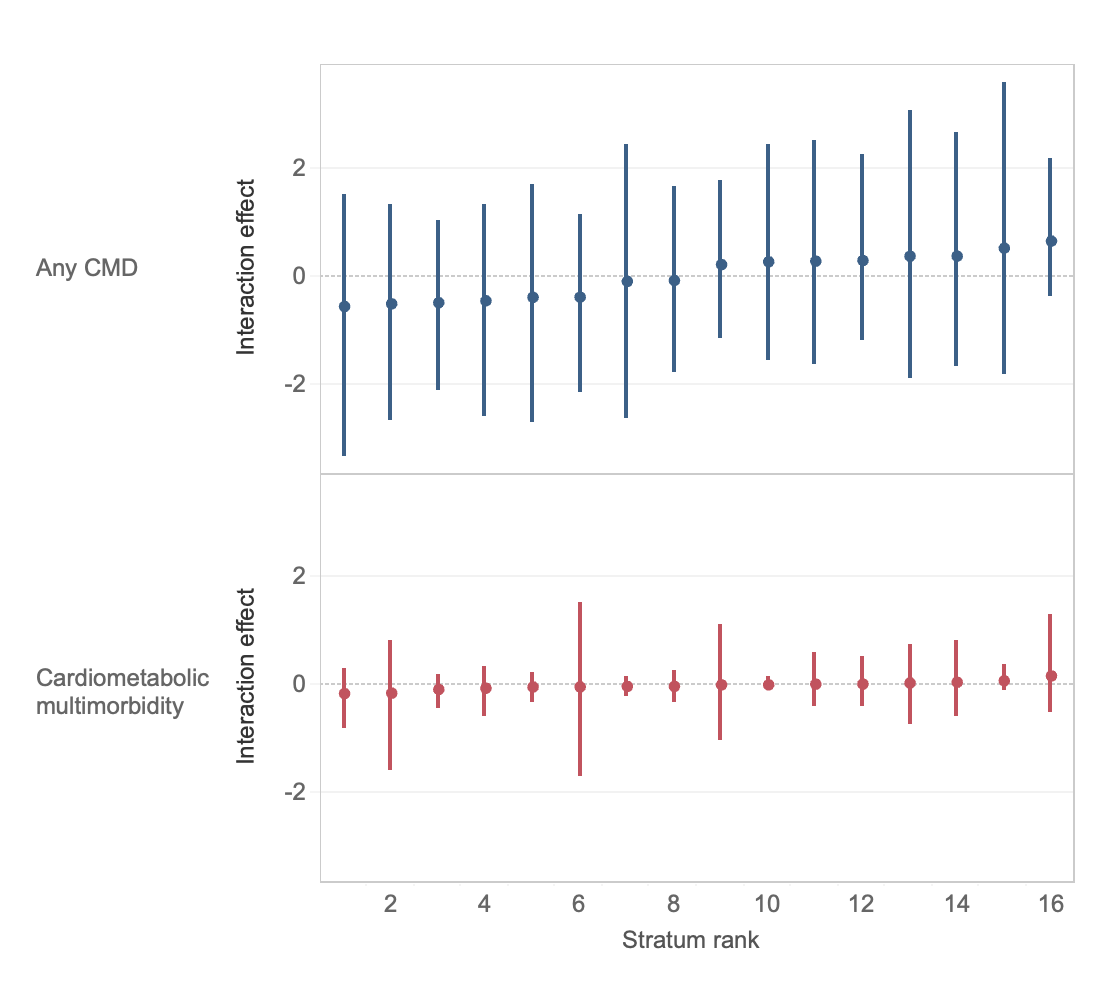


Notes:

Interaction effect ($\pi_{j}^{B}$) = predicted prevalence based on total effect ($\pi_{j}$) – predicted prevalence based on main effect ($\pi_{j}^{A})$. Numbers and stratum rank are available in Table S7.

Figure S3. Scatter plot of absolute risk (AR) and cases in the population, by social strata

Notes:

Absolute risk/prevalence was estimated by dividing the number of cases in a specific socio-geographical stratum by the stratum size. The proportion of cases in the population was estimated by dividing the number of cases in a specific socio-geographical stratum by the total number of 'any CMD' cases (n=7916) or 'cardiometabolic multimorbidity' cases (n=807). The marker size reflects the stratum size. The corresponding numbers are presented in Table S5.
